# Dual-organelle targeted photosensitizer with AIE characteristics for triple-negative breast cancer photodynamic therapy via apoptosis and immunogenic cell death

**DOI:** 10.1016/j.mtbio.2025.101828

**Published:** 2025-05-04

**Authors:** Wei Wen, Jianqing Li, Wenzhao Shang, Zeyan Zhuang, Xiepeng Deng, Xueke Yan, Dalu Xie, Chen Cui, Zujin Zhao, Ben Zhong Tang, Huifang Su

**Affiliations:** a Department of Orthopaedic Surgery, The First Affiliated Hospital of Zhengzhou University, Zhengzhou, Henan, 450052, China; b State Key Laboratory of Luminescent Materials and Devices, Guangdong Provincial Key La-boratory of Luminescence from Molecular Aggregates, South China University of Technology, Guangzhou, 510640, China; c School of Science and Engineering, Shenzhen Institute of Aggregate Science and Technology, The Chinese University of Hong Kong, Shenzhen, Guangdong, 518172, China

**Keywords:** Breast cancer, Photodynamic therapy (PDT), Apoptosis, Immunogenic cell death (ICD), Aggregation-induced emission (AIE), Dual-organelle targeting

## Abstract

Triple-negative breast cancer (TNBC) is a highly malignant breast cancer with a high metastasis rate, weak targeted therapy effect, and short patient survival period. Current treatments have significant limitations, highlighting an urgent need for novel therapies to alleviate patient suffering. Photodynamic therapy (PDT) has emerged as a promising antitumor strategy by inducing apoptosis and immune responses through the release of reactive oxygen species (ROS). However, conventional photosensitizers (PSs) face issues such as high cytotoxicity and aggregation-caused quenching (ACQ), limiting their clinical applicability. To address these challenges, we developed a novel dual-targeting, bimolecular pathway using an advanced photosensitizer, 2TPA-PIMe, designed based on aggregation-induced emission (AIE). The rationally designed 2TPA-PIMe, incorporating alkylated phosphindole as the core, exhibits AIE properties and efficient ROS generation via both type I and type II pathways, potentially enhancing PDT effectiveness *in vivo*. This approach achieves an effective dual-targeting, dual-mechanism pathway, offering new directions and methodologies for treating TNBC.

## Highlights

• New organic photosensitizer (**2TPA-PIMe**) could be simply synthesized through simple reactions, exhibiting notable characteristics such as aggregation-induced emission and excellent photostability.
• **2TPA-PIMe** has excellent type I and type II ROS generation capabilities with low-power light irradiation, which makes it possible to cure the tumor cells even with the hypoxic state of tumor tissue.
• **2TPA-PIMe** has the capability of targeting dual organelles (mitochondrial and endoplasmic reticulum) at the same time, which may augment the PDT efficiency of tumor cells as well as mouse tumor models.
• **2TPA-PIMe** possesses efficient dual tumor-killing pathways of apoptosis induction and immunogenic cell death with low-power light irradiation, which efficiently augment the killing efficiency of tumor cells.
• **2TPA-PIMe** enables good two-photon imaging under CLSM for *in vitro* samples, tissue sections, and *in vivo* applications in zebrafish, providing exceptionally clear imaging of zebrafish eyeballs under two-photon excitation, with the ability to distinguish individual live cell with a simple staining method.

## Graphical abstract

This photosensitizer (2TPA-PIMe) can target mitochondria and endoplasmic reticulum, and generate type Ⅰ and type Ⅱ ROS in light exposure, inducing apoptosis and immunogenic cell death with low power white-lightirradiation.

**Figure:**
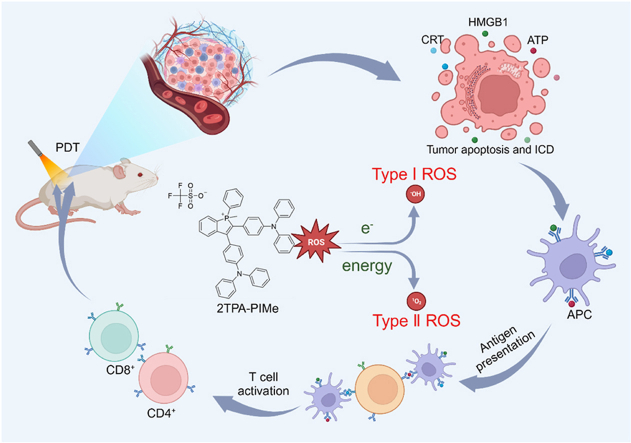

## 1 Introduction

Breast cancer is the most common malignant tumor in women. According to statistics from 2022, breast cancer in women is the second most common cancer in the world, accounting for 11.6 % [1]. For women, breast cancer is one of the most common causes of cancer death in women. Triple-negative breast cancer is known for its aggressiveness, characterized by a younger age of onset, a higher average tumor size, a higher tumor grade, and sometimes a higher rate of positive lymph nodes. Most triple-negative breast cancers are not resistant to chemotherapy. These patients have an extremely poor prognosis and will relapse and die quickly.

Photodynamic therapy (PDT) has emerged as a promising approach to tumor treatment, offering precise spatial and temporal targeting and minimal invasiveness [[2], [3], [4], [5], [6], [7], [8]]. PDT operates by releasing cytotoxic reactive oxygen species (ROS) through type I or type II reactions. These ROS quickly interact with biological molecules, inducing apoptosis, necrosis, vascular damage, and immune responses to exert antitumor effects [9,10]. A variety of photosensitizers (PSs) have been developed for PDT, including inorganic nanomaterials [11], metal-organic frameworks [12], semiconducting polymer nanoparticles [13], and pure organic molecules [14]. However, the potential biotoxicity and complex synthesis of inorganic and metallic PSs have hindered their clinical application. In contrast, pure organic PSs offer clear advantages such as well-defined structures, modifiable functional groups, and excellent biocompatibility. Despite these benefits, traditional organic PSs often suffer from aggregation-caused quenching (ACQ), poor photostability, and narrow Stokes shifts, which limit their effectiveness in clinical settings [[15], [16], [17], [18], [19], [20]]. Unlike conventional PSs, photosensitizers with aggregation-induced emission (AIE) properties exhibit stable fluorescence, enhancing their traceability and ROS generation, which in turn leads to more effective antitumor activity [[21], [22], [23], [24], [25], [26], [27]]. However, the short half-life of ROS and the fact that they can only act in the vicinity of the site of production significantly limit the efficacy of photodynamic therapy. Organelles are indispensable for the normal functioning of cells. Therefore, effective targeting of photosensitizers to the organelles is an effective strategy to enhance the efficacy of photodynamic therapy in triple-negative breast cancer [[28], [29], [30]].

**Scheme 1:**
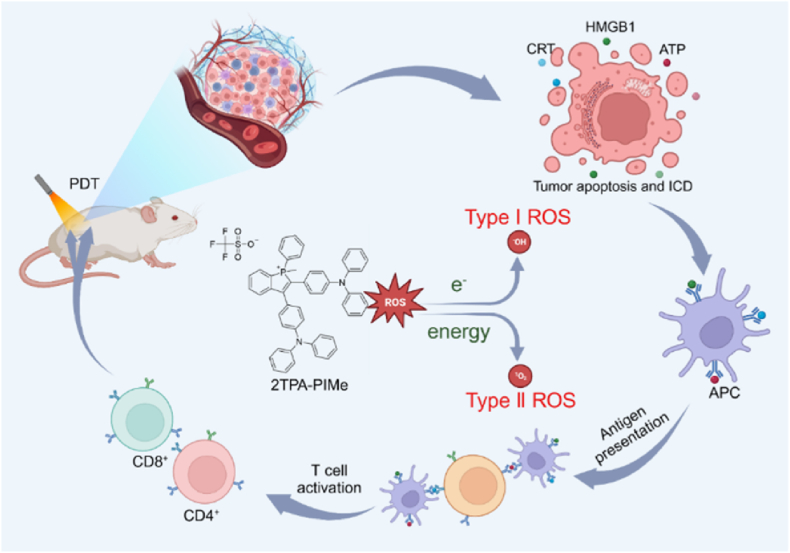
Diagram illustrating the structure of 2TPA-PIMe and the mechanism of 2TPA-PIMe-mediated dual-mode ROS activation, which induces apoptosis and immunogenic cell death.

In this study, we designed and synthesized a novel PS, 2TPA-PIMe, featuring a donor-acceptor-donor (D-A-D) structure. This PS was developed using triphenylamine (TPA) as the electron-donating (D) group and alkylated phosphindole (PIMe) as the electron-accepting (A) group, based on previous research [[31], [32], [33], [34], [35]]. The alkylated phosphindole component has a stronger electron-withdrawing capacity than the P=O structure, allowing it to act as a more potent acceptor and causing a red shift in the absorption spectrum. The 2TPA-PIMe PS demonstrates high chemical stability, photostability, and low toxicity, along with efficient ROS generation (type I and type II) under low-power white-light irradiation. Additionally, it readily crosses cell membranes, targeting both mitochondria and the endoplasmic reticulum (ER) to induce apoptosis via mitochondrial stress and stimulate robust immunogenic cell death (ICD) through ER stress. This process releases damage-associated molecular patterns (DAMPs) [36], enhancing tumor immunogenicity. In summary, 2TPA-PIMe achieves potent antitumor effects through dual ROS activation and dual-organelle targeting, representing a promising advancement in Triple-negative breast cancer treatment (see Scheme 1).

## 2 Results and discussion

**Fig. 1:**
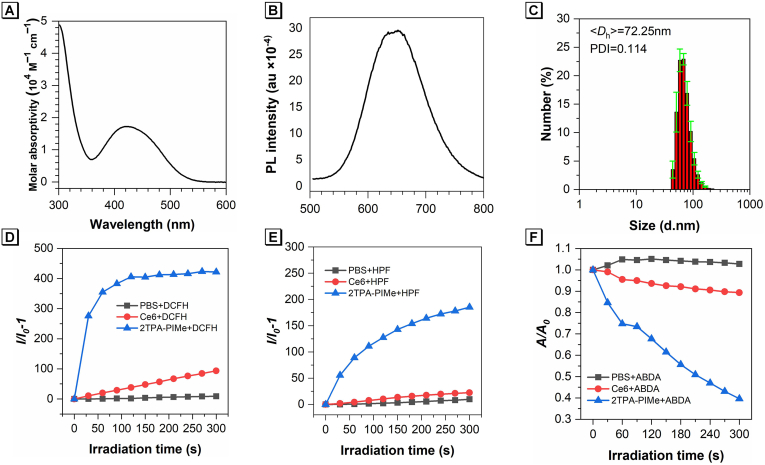
(A) Absorption spectra of 2TPA-PIMe in DMSO (10 μM). (B) PL spectra of 2TPA-PIMe in DMSO (10 μM). (C) Dynamic light scattering (DLS) plot of 2TPA-PIMe at *f*_w_ = 99 vol%. (D) Overall ROS generation upon white light irradiation (20 mW cm^−2^) using DCFH (10 μM) as an indicator. *I*_0_ and *I* represent fluorescence intensity before and after irradiation, respectively. (E) •OH generation upon white light irradiation (20 mW cm^−2^) using HPF (5 μM) as an indicator. *I*_0_ and *I* represent fluorescence intensity before and after irradiation, respectively. (F) ^1^O_2_ generation upon white light irradiation (20 mW cm^−2^) using ABDA as an indicator. *A*_0_ and *A* denote ABDA absorbance (10 μM) before and after irradiation, respectively.

**Photophysical and photodynamic properties.** The synthesis of 2TPA-PIMe was first characterized by the C-H spectrum as well as the mass spectrum (Fig. S1). The absorption and photoluminescence (PL) spectra of 2TPA-PIMe were initially measured to evaluate its optical properties. In dimethyl sulfoxide (DMSO), 2TPA-PIMe exhibited a maximum absorption peak at 435 nm and a fluorescence peak at 650 nm (Fig. 1A and B). The molecule displayed a large Stokes shift (∼220 nm), attributed to its multiple rotors, which effectively reduces background fluorescence interference and enhances the sensitivity of biological imaging. Due to its good solubility in DMSO and insolubility in water, the aggregation-induced emission (AIE) properties of 2TPA-PIMe were assessed by varying water fractions (*f*_w_) in DMSO-water mixtures. As shown in Fig. S2, adding water initially decreased fluorescence intensity, likely due to the twisted intramolecular charge transfer (TICT) effect [37,38]. However, as water content increased beyond 60 %, fluorescence intensity rose, suggesting the dominance of the AIE effect. Dynamic light scattering (DLS) analysis revealed the formation of nanoaggregates at *f*_w_ = 99 %, with an average hydrodynamic diameter (*D*_h_) of 72.25 nm (Fig. 1C), confirmed through transmission electron microscopy (TEM) imaging (Fig. S3).

To further investigate 2TPA-PIMe's potential as a photosensitizer, its ROS production capability in aqueous solution was evaluated under white light irradiation. Using 2′,7′-Dichlorodihydrofluorescein (DCFH) as a ROS indicator, the generation efficiency was measured based on fluorescence at 525 nm [39]. As shown in Fig. 1D, upon white light irradiation at 20 mW cm^−2^, the fluorescence intensity of DCFH in the presence of 1 μM 2TPA-PIMe increased approximately 165-fold within 5 min, indicating highly efficient ROS generation. In contrast, the fluorescence intensity in the PBS control group remained nearly unchanged under identical conditions.

Depending on ROS generation mechanisms, PDT can be classified as type I or type II. Type I PDT involves electron transfer, generating free radicals, while type II produces singlet oxygen (^1^O_2_) through energy transfer. Type I PDT is less dependent on oxygen, making it advantageous in the hypoxic tumor microenvironment. Even under severe oxygen depletion (2 % O_2_), ROS such as hydroxyl radicals (•OH) can be generated through disproportionation and Haber-Weiss/Fenton reactions, effectively targeting tumor cells [[40], [41], [42]].

To analyze the specific ROS generated by 2TPA-PIMe, Hydroxyphenyl Fluorescein (HPF) and 9,10-Anthracenediyl-bis(methylene) dimalonic acid (ABDA) were employed to detect •OH and ^1^O_2_, respectively [[43], [44], [45]]. As shown in Fig. 1E, in the presence of 1 μM 2TPA-PIMe under white light irradiation (20 mW cm^−2^) for 5 min, the emission intensity of HPF increased fourfold, indicating •OH production. By contrast, the PBS control showed minimal fluorescence change under the same conditions. Simultaneously, the absorption intensity of ABDA in 2TPA-PIMe solutions decreased by about 33 %, while the PBS control group showed no significant change (Fig. 1F). These findings indicate that 2TPA-PIMe can efficiently generate both type I and type II ROS, making it a highly suitable candidate for PDT in tumor treatment.

**Fig. 2:**
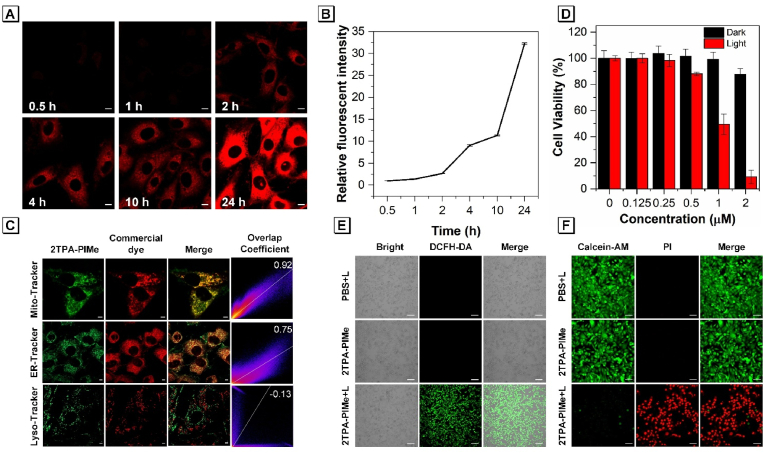
(A) Uptake of 2TPA-PIMe by MG-63 cells and (B) relative fluorescence quantification after various incubation times. Scale bar: 10 μm. (C) Co-localization images of MG-63 cells with 2TPA-PIMe (1 μM) and Mito-Tracker Red, ER-Tracker Red, and Lyso-Tracker Red. Scale bar: 5 μm. (D) Cell viability of MG-63 cells after treatment with different concentrations of 2TPA-PIMe, with or without white light irradiation. (E) Live/dead staining assay of MG-63 cells and (F) general ROS generation of 2TPA-PIMe in MG-63 cells following treatments with PBS + Light (PBS + L), 2TPA-PIMe, and 2TPA-PIMe + Light (2TPA-PIMe + L). (White light: 40 mW cm^−2^ for 10 min). Scale bar: 50 μm.

**Cell imaging.** To assess the biological potential of 2TPA-PIMe in tumor imaging and therapy, we first explored its uptake by cancer cells. MG-63 cells were co-incubated with 2TPA-PIMe (1 μM) for varying times (0.5, 1, 2, 4, 10, and 24 h), followed by confocal laser scanning microscopy (CLSM) imaging. Fluorescent signals were detected as early as 0.5 h, indicating excellent cell penetration. Additionally, the fluorescence intensity within the cells progressively increased with longer incubation times, demonstrating time-dependent uptake (Fig. 2A and B).

Next, we investigated the subcellular localization of 2TPA-PIMe in MG-63 cells by co-incubating with commercial organelle dyes (Mito-Tracker Red, ER-Tracker Red, and Lyso-Tracker Green) and imaging with CLSM. As shown in Fig. 2C, 2TPA-PIMe primarily localized in the mitochondria and ER of MG-63 cells, with the Pearson co-localization coefficients of 92 % and 75 %, respectively, as indicated by overlapping red and green fluorescence signals. This dual-targeting was similarly observed in 4T1 cells (Fig. S4 and S5), suggesting that 2TPA-PIMe could serve as an effective probe for targeting mitochondria and ER, thus supporting its potential in dual-targeted tumor cell killing. There is a vast number of organelle-targeting photosensitizers, while dual-targeting photosensitizers are relatively rare. Zhao's group discovered a near-infrared photosensitizer that targets both the plasma membrane and mitochondria [[46], [47], [48], [49]]. 2TPA-PIMe is one of these rare photosensitizers, capable of dual targeting the endoplasmic reticulum and mitochondria.

Also, given the limited penetration of white light, and the fact that two-photon excitation utilizes the simultaneous absorption of two low-energy photons to excite a molecule, long wavelengths of light are typically used. These long wavelengths of light are less scattered and absorbed in tissues, thus enabling deeper penetration into tissues, providing higher spatial resolution and deeper imaging depth. Thus, we further validated 2TPA-PIMe two-photon imaging capability. Firstly, we stained zebrafish embryos using 2TPA-PIME, which was observed to stain multiple organs and tissues of zebrafish embryos, especially the eyes, using single-photon imaging (Fig. S6). Then we used two-photon CLSM at 850 nm to image cells, kidney tissues, and zebrafish embryos incubated with 2TPA-PIMe. The two-photon CLSM images confirmed that 2TPA-PIMe provides excellent imaging performance *in vitro*, in tissue sections, and *in vivo* (Fig. S7). Notably, two-photon CLSM revealed intricate details of zebrafish eyeballs at the single-cell level, underscoring the high-resolution imaging potential of 2TPA-PIMe. Meanwhile, by using two-photon imaging in a zebrafish model, the excitation and imaging capabilities of 2TPA-PIMe in deep tissues can be verified. This provides an important experimental basis for future application of two-photon excitation in more complex biological models such as mammals.

**Photodynamic therapy of 2TPA-PIMe *in vitro*.** To assess the feasibility of 2TPA-PIMe in biological applications, its ROS-generating capability under white light irradiation was tested in MG-63 and 4T1 cells using DCFH-DA as a general ROS indicator, visualized by CLSM. As shown in Fig. 2E and S10A, after incubation with 1 μM 2TPA-PIMe for 4 h and subsequent irradiation with 40 mW cm^−2^ white light for 5 min, intracellular green fluorescence was significantly increased (2TPA-PIMe + L), while there was almost no fluorescence signal in the PBS + L and 2TPA-PIMe-only groups, confirming that 2TPA-PIMe efficiently generates ROS under white light.

The dark and photo-cytotoxicity of 2TPA-PIMe were then evaluated using MTT assays to examine its biocompatibility and photodynamic efficiency. After incubating MG-63 or 4T1 cells with different concentrations of 2TPA-PIMe for 4 h, cells were irradiated with 40 mW cm^−2^ white light for 10 min, followed by 20 h of incubation in the dark. Control cells were kept in the dark for 24 h. As shown in Fig. 2D and S8, 2TPA-PIMe exhibited negligible cytotoxicity in the absence of light, demonstrating good biocompatibility. Under light exposure, cell viability decreased significantly with increasing concentrations of 2TPA-PIMe, with over 50 % cell death at 1 μM and more than 90 % at 2 μM, indicating strong PDT efficacy. Meanwhile, we co-incubated with vitamin C and 2TPA, and after white light irradiation, the cell viability was significantly higher than before (Fig. S9).

To further illustrate the PDT effect, Calcein-AM/Propidium Iodide (PI) co-staining was performed. In this method, live cells are stained with Calcein-AM (green), while dead cells are labeled with PI (red), and the cells are imaged by CLSM. As shown in Fig. 2F and S10B, cells in the PBS + L and 2TPA-PIMe-only groups (1 μM) displayed green fluorescence, indicating intact cell membranes and high cell viability. In contrast, the 2TPA-PIMe + L group (40 mW cm^−2^) exhibited extensive red fluorescence, indicating membrane rupture and cell death. These results confirm the potent *in vitro* antitumor effects of 2TPA-PIMe.

**Fig. 3:**
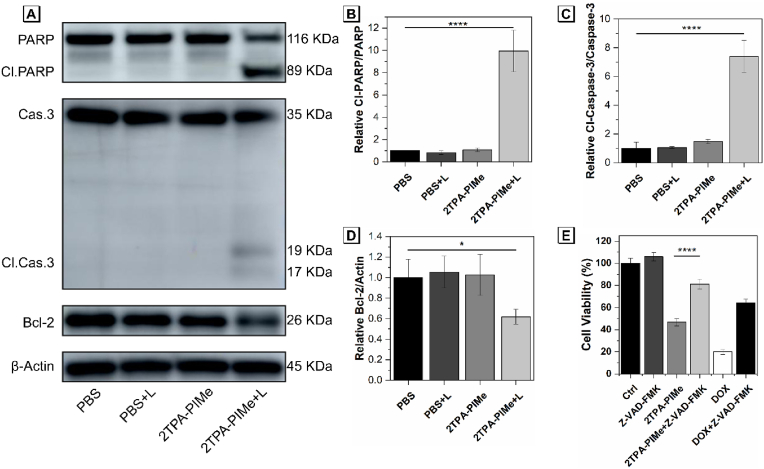
(A) Western blot analysis of apoptosis-related proteins in MG-63 cells treated with PBS or 2TPA-PIMe for 4 h, with or without white light irradiation (40 mW cm^−2^) for 10 min. (B), (C), and (D) Quantitative analyses of relative protein expression after different treatments. (E) Cell viability of MG-63 cells after treatment with 2TPA-PIMe + light (with or without apoptosis inhibitor Z-VAD-FMK) and doxorubicin (DOX) as a control. Data are shown as mean ± SD, n ≥ 3 (*p* < 0.05, ∗; *p* < 0.01, ∗∗; *p* < 0.001, ∗∗∗; *p* < 0.0001, ∗∗∗∗; ns, not significant).

Based on co-localization analysis, 2TPA-PIMe primarily accumulates in mitochondria and ER, leading to localized photodamage to these organelles due to the limited diffusion range of ROS [[46], [47], [48]]. We further investigated the cytological mechanisms underlying 2TPA-PIMe-induced PDT cell death, focusing on mitochondria-mediated apoptosis. Apoptosis is a programmed cell death process that plays a key role in maintaining tissue homeostasis and removing damaged or abnormal cells. In tumor therapy, apoptosis can help remove damaged cells or potential cancer cells and maintain tissue homeostasis. When a cell is stimulated by DNA damage, oxidative stress, etc., if the damage cannot be repaired, the cell will initiate the apoptotic program to prevent these damaged cells from continuing to proliferate and develop into tumors. At the same time, apoptosis can release tumor antigens, activate the immune system, and enhance the ability of immune cells to recognize and kill tumors, thus improving the therapeutic effect. Apoptosis can be activated through multiple pathways, so even if tumor cells become resistant to a certain treatment, inducing apoptosis through other pathways may still be effective, thus reducing the impact of drug resistance on the therapeutic effect. Therefore, by inducing apoptosis in tumor cells, the growth and spread of tumors can be effectively inhibited. Western blot analysis was conducted to examine the expression of proteins involved in mitochondria-mediated apoptosis during 2TPA-PIMe PDT (Fig. 3A). B-cell lymphoma 2 (Bcl-2), an anti-apoptotic protein located in the mitochondrial membrane, prevents the release of pro-apoptotic factors by regulating mitochondrial permeability. Under stress, Bcl-2 expression decreases, leading to increased mitochondrial membrane permeability and the release of pro-apoptotic factors that activate caspase-3, initiating a caspase cascade that cleaves intracellular substrates, such as PARP, inhibits DNA repair, and triggers DNA degradation, ultimately leading to apoptosis.

MG-63 cells were incubated with 2 μM 2TPA-PIMe for 4 h, irradiated with 40 mW cm^−2^ white light for 10 min, and then further incubated for 4 h. As shown in Fig. 3B–D, in the 2TPA-PIMe group, Bcl-2 expression was reduced, caspase-3 was activated with cleaved caspase-3 production, and PARP was degraded, indicating that 2TPA-PIMe-induced PDT promotes apoptosis. This conclusion was supported by MTT assays. MG-63 cells were pre-incubated with or without the apoptosis inhibitor Z-VAD-FMK for 2 h, followed by co-incubation with 1 μM 2TPA-PIMe. After 4 h, cells were exposed to 40 mW cm^−2^ white light for 10 min and then incubated in the dark for 20 h. Cell viability in the 2TPA-PIMe + light group decreased to below 50 %, while the presence of Z-VAD-FMK increased viability by approximately 30 % (*p* < 0.0001), further supporting apoptosis as the primary mode of cell death. A control experiment using doxorubicin, an apoptosis-inducing chemotherapeutic drug, showed similar results (Fig. 3E), confirming that apoptosis was induced by PDT.

**Fig. 4:**
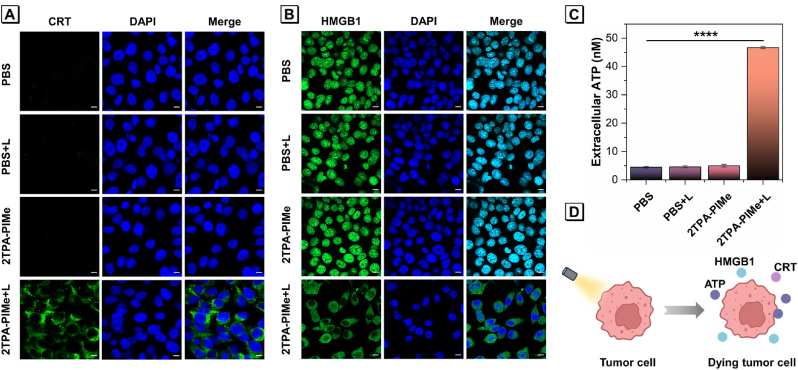
Immunofluorescence images of (A) CRT and (B) HMGB1 in MG-63 cells following treatment with PBS or 2TPA-PIMe (1 μM), with or without white light irradiation (40 mW cm^−2^) for 10 min, followed by a 4-h incubation. Scale bar: 10 μm. (C) Quantification of extracellular adenosine triphosphate (ATP) in MG-63 cells treated with PBS or 2TPA-PIMe (1 μM), with or without irradiation (40 mW cm^−2^). Data are presented as mean ± SD, n ≥ 3 (*p* < 0.05, ∗; *p* < 0.01, ∗∗; *p* < 0.001, ∗∗∗; *p* < 0.0001, ∗∗∗∗; not significant, ns). (D) Schematic illustration of ICD effects in cancer cells, characterized by CRT exposure, ATP secretion, and HMGB1 release.

Simultaneously, given the high accumulation of 2TPA-PIMe in the ER, we examined its potential to induce immunogenic cell death (ICD) through ER stress. ICD is a promising antitumor strategy that promotes tumor suppression by activating both innate and adaptive immunity through damage-associated molecular patterns (DAMPs) such as calreticulin (CRT), high mobility group box-1 protein (HMGB1), and adenosine triphosphate (ATP) (Fig. 4D) [36]. To investigate this, MG-63 and 4T1 cells were treated with PBS, 2TPA-PIMe alone, PBS with light irradiation, and 2TPA-PIMe with light irradiation. DAMP expression was then evaluated using immunofluorescence imaging via CLSM.

As shown in Fig. 4A and S11A, the 2TPA-PIMe + light (L) group exhibited a significant increase in green fluorescence for CRT in both the cytoplasm and cell membrane. CRT, a protein normally residing in the ER, translocates to the cell membrane under stress, where it serves as an "eat-me" signal to immune cells, initiating an immune response [50,51]. In contrast, cells in the other three groups displayed negligible green fluorescence expression. HMGB1, another DAMP, acts as a danger signal for pattern recognition receptors on antigen-presenting and other immune cells. During ICD, HMGB1 migrates from the nucleus to the cytoplasm and is eventually released extracellularly, where it activates immune signaling pathways [52]. As shown in Fig. 4B and S11B, green fluorescence was localized within the nucleus in cells treated with PBS, PBS + L, and 2TPA-PIMe alone, whereas in the 2TPA-PIMe + L group, the fluorescence had shifted almost entirely from the nucleus to the pericellular region.

Additionally, extracellular ATP levels in the 2TPA-PIMe + L group increased approximately 9-fold and 4.3-fold compared to the other three groups (Fig. 4C and S11C). During ICD, ATP is released extracellularly, serving as a "find-me" signal that encourages phagocytes to target and engulf tumor cells [51,[53], [54], [55]]. These findings strongly suggest that the 2TPA-PIMe-mediated photodynamic therapy process effectively induces ICD effects. In addition, the immunofluorescence of HSP70 also confirms that the target molecule induces cells to generate immunogenic cell death (ICD) under light irradiation (Fig. S12).

**Fig. 5:**
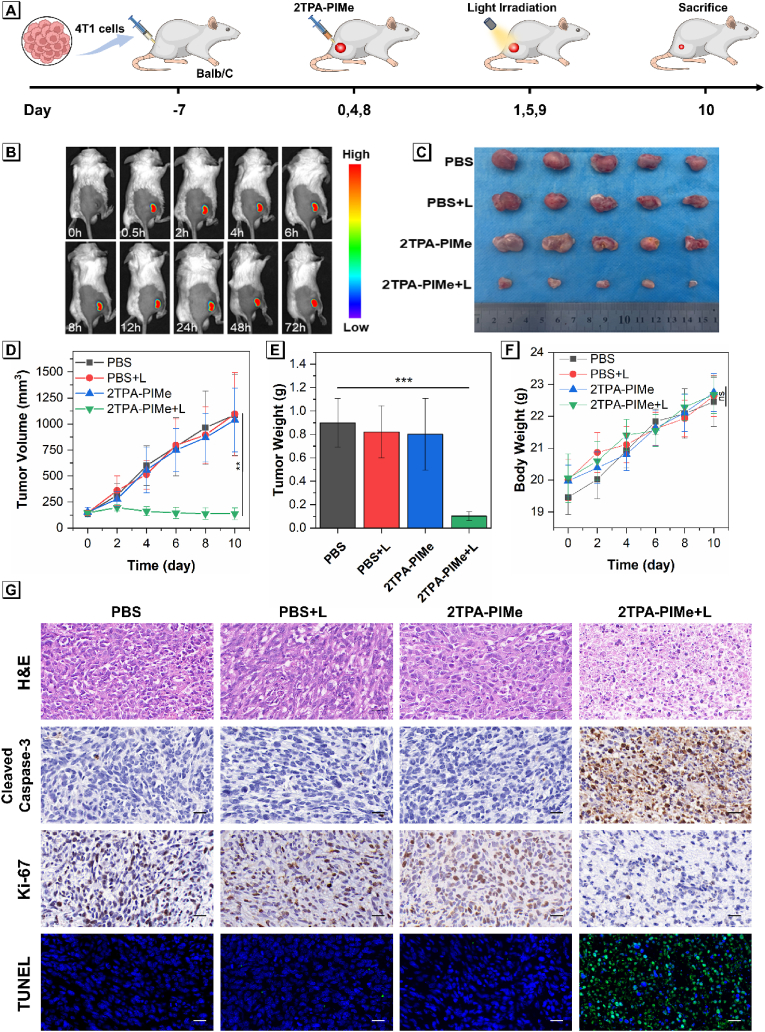
(A) Schematic illustration of the construction of the subcutaneous 4T1 tumor model and *in vivo* antitumor therapy in Balb/C mice. (B) Fluorescence images of mouse tumors at various time points following intratumoral injection of 2TPA-PIMe. (C) Photographs of resected orthotopic 4T1 tumors in different treatment groups (n = 5). (D) Subcutaneous 4T1 tumor growth curves across treatment groups. (E) Subcutaneous 4T1 tumor weight across treatment groups. (F) Body weight changes in subcutaneous 4T1 tumor-bearing mice across treatment groups. Data presented as mean ± SD, n = 5 (*p* < 0.05, ∗; *p* < 0.01, ∗∗; *p* < 0.001, ∗∗∗; *p* < 0.0001, ∗∗∗∗; not significant, ns). (G) H&E staining, immunohistochemistry, and immunofluorescence images (Cleaved Caspase-3, Ki-67, and TUNEL) of tumor tissues after different treatments. Scale bar: 20 μm.

***In vivo* imaging and photodynamic therapy evaluation of 2TPA-PIMe.** Based on the promising cellular performance of 2TPA-PIMe, we selected the 4T1 breast cancer cell line as a model for *in vivo* experiments. Using a subcutaneous tumor-bearing mouse model, we evaluated the PDT efficacy of 2TPA-PIMe. First, we assessed its imaging capabilities within the tumor. Once the tumor volume reached approximately 150 mm^3^, we initiated *in vivo* experiments. Following the intratumoral injection of 2TPA-PIMe (5 mg kg^−1^, 50 μL), bright fluorescence signals were observed at the tumor site within 0.5 h and remained stable for up to 72 h without spreading to surrounding normal tissues, demonstrating 2TPA- PIMe's strong tumor retention and its potential for tumor imaging (Fig. 5B).

Next, we evaluated the antitumor efficacy of 2TPA-PIMe-mediated PDT *in vivo*. Mice were randomly divided into four gro [1]ups: PBS, PBS with white light (PBS + L), 2TPA-PIMe, and 2TPA-PIMe with white light (2TPA-PIMe + L). As shown in Fig. 5A, when tumors reached ∼150 mm^3^, mice received intratumoral injections of either 2TPA-PIMe (5 mg kg^−1^, 50 μL) or PBS (50 μL). Considering that significant killing effects can be achieved in cell experiments when the drug is co-cultured with cells for 24 h before light exposure, the 24-h incubation process was chosen to be applied to animal treatment. The mice in the PBS + L and 2TPA-PIMe + L groups were then exposed to white light (200 mW cm^−2^) at the tumor site for 30 min. Although 2TPA-PIMe demonstrated extended tumor retention, intratumoral injections were administered every three days to optimize therapeutic results, with the treatment repeated three times over the experiment's duration. Tumor volume and body weight were monitored every two days, with tumors dissected and weighed at the study's conclusion.

As shown in Fig. 5C and D, tumors in the PBS, PBS + L, and 2TPA-PIMe groups grew rapidly, reaching nearly 1000 mm^3^ within 11 days. In contrast, tumor growth in the 2TPA-PIMe + L group was significantly suppressed and even slightly reduced, with a fivefold reduction in tumor volume (*p* < 0.01). Furthermore, as depicted in Fig. 5E, the PBS group exhibited a ninefold increase in tumor mass compared to the 2TPA-PIMe + L group (*p* < 0.001), highlighting the substantial antitumor efficacy of 2TPA-PIMe-mediated PDT. Importantly, body weights across all groups remained stable, with no significant weight loss observed, suggesting good biocompatibility of 2TPA-PIMe (*p* > 0.05, Fig. 5F).

Histological analysis provided further evidence of 2TPA-PIMe's effectiveness. Hematoxylin and eosin (H&E) staining of tumors from the 2TPA-PIMe + L group showed significant vacuolization, numerous missing nuclei, and notable nuclear condensation, indicating effective tumor destruction by PDT. Additionally, terminal deoxynucleotidyl transferase dUTP nickend labeling (TUNEL) and cleaved caspase-3 staining confirmed that 2TPA-PIMe under white light induced extensive tumor apoptosis (Apoptotic cells are marked in green). Immunohistochemical analysis of Ki-67 also demonstrated marked inhibition of tumor proliferation in the 2TPA-PIMe + L group, while the other groups displayed densely packed tumor cells with high proliferative activity (Fig. 5G).

To confirm whether the enhanced antitumor effect observed *in vivo* was immune-related, DAMPs were initially examined. Consistent with cellular experiment data, the 2TPA-PIMe + L group exhibited the highest levels of CRT expression and the most significant HMGB1 translocation and release (red fluorescence), compared to the three control groups. The released DAMPs play a crucial role in recruiting and maturing antigen-presenting cells, which subsequently activate T cells.

**Fig. 6:**
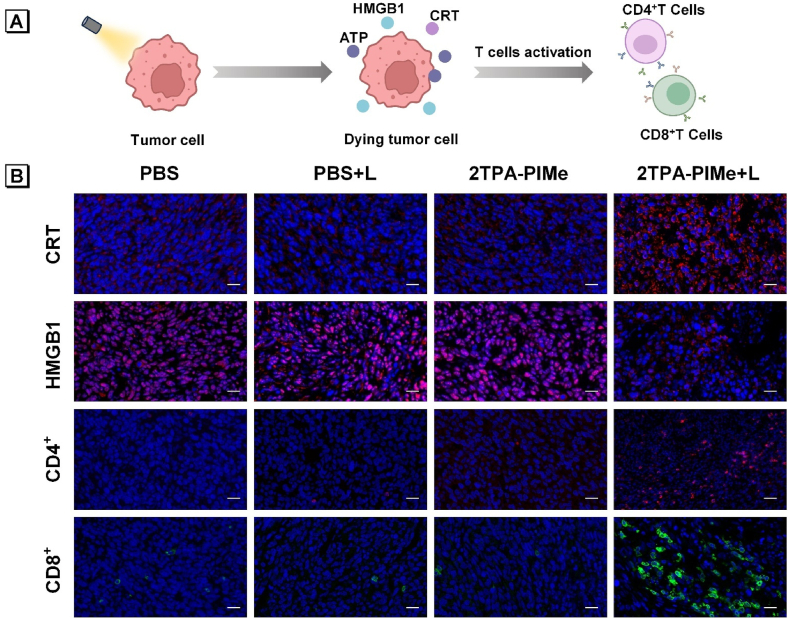
(A) Schematic illustration showing the mechanism by which 2TPA-PIMe achieves ICD-based photodynamic immunotherapy. (B) Immunofluorescence imaging of CRT, HMGB1, CD4^+^, and CD8^+^ staining in tumor tissues after various treatments. Scale bar: 20 μm.

To detect T cell activation in ICD, mouse tumors were excised and subjected to immunofluorescence staining. CD8^+^ T cells, known as key drivers of antitumor immunity, can mediate tumor rejection by recognizing tumor antigens and directly killing cancerous cells. Additionally, CD4^+^ T cells are often required to support CD8^+^ T cell activation, providing co-stimulatory signals for optimal immune response. In the 2TPA-PIMe + L group, significant fluorescence signals from CD4^+^ T (red) and CD8^+^ T (green) cells indicated substantial T-cell infiltration in the tumor tissues, suggesting effective immune activation upon photodynamic treatment. (Fig. 6B).

These findings clearly demonstrate that 2TPA-PIMe-mediated PDT induces apoptosis, inhibits tumor cell proliferation, and promotes immunogenic cell death, thereby achieving potent antitumor effects (Fig. 6A).

***In vivo* toxicity assay.** To assess the biosafety of 2TPA-PIMe, an *in vivo* toxicity evaluation was conducted, addressing the importance of minimizing systemic toxicity for clinical applications. After 11 days of treatment with 2TPA-PIMe, mice were sacrificed, and blood samples were collected for biochemical analysis. As shown in Fig. S10, all measured biochemical parameters—alanine aminotransferase (ALT), total bilirubin (TBIL), alkaline phosphatase (ALP), albumin (ALB), blood urea nitrogen (BUN), creatinine (CREA), and uric acid (UA)—remained within normal ranges, with no significant differences observed across treatment groups, indicating negligible hepatotoxicity and nephrotoxicity *in vivo*.

Furthermore, routine blood tests revealed that after treatment with 2TPA-PIMe + light exposure (L), all white blood cell indicators in mice were close to normal levels. In contrast, the other three groups showed abnormal white blood cell indicators due to advanced tumor progression (Fig. S13 and Table S1). Major organs, including the heart, liver, spleen, lungs, and kidneys, were also collected from each group for histological analysis. Hematoxylin and eosin (H&E) staining showed no visible tissue damage or inflammatory changes in any organ (Fig. S14). These findings collectively demonstrate the biocompatibility and potential for biological applications of 2TPA-PIMe *in vivo*.

## 3 Conclusion

In summary, we have successfully developed an effective photosensitizer (2TPA-PIMe) with AIE properties, utilizing an alkylated phosphindole core. Compared with general photosensitizers, it can be synthesized by a simple reaction and has remarkable properties such as aggregation-induced emission and excellent photostability. Our findings demonstrate that 2TPA-PIMe can spontaneously form nanoparticles in aqueous solution that exhibit robust type I and type II ROS generation, which makes it possible to cure tumor cells even when the tumor tissue is hypoxic. Both *in vivo* and *in vitro* experiments confirmed its strong fluorescence imaging ability and dual targeting of mitochondria and ER, resulting in high phototoxicity and apoptosis induction. Furthermore, 2TPA-PIMe enhances tumor immunogenicity, activates immune responses, promotes T-cell infiltration, and achieves significant antitumor effects. Organ histology and blood biochemistry analyses further validate its excellent biocompatibility and low systemic toxicity.

A known limitation of conventional photosensitizers is their reliance on visible light for excitation, which suffers from limited wavelength and poor penetration depth, reducing PDT effectiveness in treating deep-seated tumors. Notably, 2TPA-PIMe can be excited by a two-photon laser at 850 nm, providing improved tissue penetration. Further research will focus on exploring two-photon excited PDT to enhance therapeutic outcomes for tumors. Besides, 2TPA-PIMe enables good two-photon imaging under CLSM for *in vitro* samples, tissue sections, and *in vivo* applications in zebrafish, providing exceptionally clear imaging of zebrafish eyeballs under two-photon excitation, with the ability to distinguish individual live cells with simple staining method, which may make it possible to achieve two-photon excited imaging and photodynamic therapy in cancer research.

Overall, this study introduces a high-performance photosensitizer with dual organelle-targeting capabilities and dual-mode ROS activation. 2TPA-PIMe offers a potent photodynamic approach for inducing apoptosis and immunogenic cell death, presenting a promising treatment for Triple-negative breast cancer and other malignancies.

## CRediT authorship contribution statement

**Wei Wen:** Writing – original draft, Methodology, Investigation, Formal analysis, Data curation. **Jianqing Li:** Writing – original draft, Validation, Methodology, Investigation, Formal analysis, Data curation. **Wenzhao Shang:** Writing – original draft, Methodology, Investigation. **Zeyan Zhuang:** Methodology. **Xiepeng Deng:** Methodology. **Xueke Yan:** Methodology. **Dalu Xie:** Methodology. **Chen Cui:** Methodology. **Zujin Zhao:** Writing – review & editing, Supervision, Project administration, Funding acquisition, Conceptualization. **Ben Zhong Tang:** Supervision, Resources. **Huifang Su:** Writing – review & editing, Supervision, Resources, Project administration, Funding acquisition, Formal analysis, Conceptualization.

## Declaration of competing interest

The authors declare that they have no conflict of interest.

## Data Availability

Data will be made available on request.

## References

[1] Bray F., Laversanne M., Sung H., Ferlay J., Siegel R.L., Soerjomataram I., Jemal A. (2024). Global cancer statistics 2022: GLOBOCAN estimates of incidence and mortality worldwide for 36 cancers in 185 countries. CA Cancer J. Clin..

[2] Sun J., Xing F., Braun J., Traub F., Rommens P.M., Xiang Z., Ritz U. (2021). Progress of phototherapy applications in the treatment of bone cancer. Int. J. Mol. Sci..

[3] Dai J., Xue H., Chen D., Lou X., Xia F., Wang S. (2022). Aggregation-induced emission luminogens for assisted cancer surgery. Coord. Chem. Rev..

[4] Wang X., Tang Y., Li Y., Qi Z. (2024). A pyroptosis-inducing arsenic(III) nanomicelle platform for synergistic cancer immunotherapy. Adv. Healthc. Mater..

[5] Wang X., Li Y., Hasrat K., Yang L., Qi Z. (2023). Sequence-Responsive multifunctional supramolecular nanomicelles act on the regression of TNBC and its lung metastasis via synergic pyroptosis-mediated immune activation. Small.

[6] Wang X., Yang L., Li Y., Wang X., Qi Z. (2024). A long-retention cell membrane-targeting AIEgen for boosting tumor theranostics. Chem. Asian J..

[7] Wang X., Li Y., Qi Z. (2024). Light-enhanced tandem-responsive nano delivery platform for amplified anti-tumor efficiency. Chem. Asian J..

[8] Wang X., Liang J., Zhao Y., Yang L., Qi Z., Tang Y. (2023). A lipid droplet-specific near-infrared automatic oxygen-supplied AIEgen for photodynamic therapy and metastasis inhibition of hypoxic tumors. Chem. Eng. J..

[9] Dolmans D.E., Fukumura D., Jain R.K. (2003). Photodynamic therapy for cancer. Nat. Rev. Cancer.

[10] Korbelik M. (2011). Cancer vaccines generated by photodynamic therapy. Photochem. Photobiol. Sci..

[11] Yu X., Gao D., Gao L., Lai J., Zhang C., Zhao Y., Zhong L., Jia B., Wang F., Chen X., Liu Z. (2017). Inhibiting metastasis and preventing tumor relapse by triggering host immunity with tumor-targeted photodynamic therapy using photosensitizer-loaded functional nanographenes. ACS Nano.

[12] Lan G., Ni K., Xu Z., Veroneau S.S., Song Y., Lin W. (2018). Nanoscale metal–organic framework overcomes hypoxia for photodynamic therapy primed cancer immunotherapy. J. Am. Chem. Soc..

[13] Li J., Pu K. (2020). Semiconducting polymer nanomaterials as near-infrared photoactivatable protherapeutics for cancer. Acc. Chem. Res..

[14] Zheng X., Wu W., Zheng Y., Ding Y., Xiang Y., Liu B., Tong A. (2021). Organic nanoparticles with persistent luminescence for in vivo afterglow imaging-guided photodynamic therapy. Chem. Eur J..

[15] Mei J., Hong Y.N., Lam J.W.Y., Qin A.J., Tang Y.H., Tang B.Z. (2014). Aggregation-induced emission: the whole is more brilliant than the parts. Adv. Mater..

[16] Mei J., Leung N.L.C., Kwok R.T.K., Lam J.W.Y., Tang B.Z. (2015). Aggregation-induced emission: together we shine, united we soar. Chem. Rev..

[17] Qian J., Tang B.Z. (2017). AIE luminogens for bioimaging and theranostics: from organelles to animals. Chem.

[18] Gao M., Su H.F., Li S.W., Lin Y.H., Ling X., Qin A.J., Tang B.Z. (2017). An easily accessible aggregation-induced emission probe for lipid droplet-specific imaging and movement tracking. Chem. Commun..

[19] Su H.-F., Peng Q.-C., Liu Y.U., Xie T., Liu P.-P., Cai Y.-C., Wen W., Yu Y.-H., Li K., Zang S.-Q. (2022). A near-infrared AIE probe and its applications for specific in vitro and in vivo two-photon imaging of lipid droplets. Biomaterials.

[20] Wang D., Su H.F., Kwok R.T.K., Shan G.G., Leung A.C.S., Lee M.M.S., Sung H.H.Y., Williams I.D., Lam J.W.Y., Tang B.Z. (2017). Facile synthesis of red/NIR AIE luminogens with simple structures, bright emissions, and high photostabilities, and their applications for specific imaging of lipid droplets and image-guided photodynamic therapy. Adv. Funct. Mater..

[21] Dai J., Wu X., Ding S., Lou X., Xia F., Wang S., Hong Y. (2020). Aggregation-induced emission photosensitizers: from molecular design to photodynamic therapy. J. Med. Chem..

[22] Dai J., Li Y., Long Z., Jiang R., Zhuang Z., Wang Z., Zhao Z., Lou X., Xia F., Tang B.Z. (2020). Efficient near-infrared photosensitizer with aggregation-induced emission for imaging-guided photodynamic therapy in multiple xenograft tumor models. ACS Nano.

[23] Xia Q., Zhang Y., Li Z., Hou X., Feng N. (2019). Red blood cell membrane-camouflaged nanoparticles: a novel drug delivery system for antitumor application. Acta Pharm. Sin. B.

[24] Dai J., Dong X., Wang Q., Lou X., Xia F., Wang S. (2021). PEG-polymer encapsulated aggregation-induced emission nanoparticles for tumor theranostics. Adv. Healthc. Mater..

[25] Kang M., Zhang Z., Song N., Li M., Sun P., Chen X., Wang D., Tang B.Z. (2020). Aggregation-enhanced theranostics: AIE sparkles in biomedical field. Aggregate.

[26] Qi J., Ou H., Liu Q., Ding D. (2021). Gathering brings strength: how organic aggregates boost disease phototheranostics. Aggregate.

[27] Du X., Liu X., Su H., Cheng X., Li L., Gu H., Xing X., Qiu D., Hao H. (2022). A simple AIEgen photosensitizer with cucurbit[7]uril selective detection amantadine and application in mitochondrion imaging. Microchem. J..

[28] Ma W., Sun R., Tang L., Li Z., Lin L., Mai Z., Chen G., Yu Z. (2023). Bioactivable sting nanoagonists to synergize Nir-Ii mild photothermal therapy primed robust and long-term anticancer immunity. Adv. Mater..

[29] Hu D., Li Y., Li R., Wang M., Zhou K., He C., Wei Q., Qian Z. (2024). Recent advances in reactive oxygen species (ROS)-responsive drug delivery systems for photodynamic therapy of cancer. Acta Pharm. Sin. B.

[30] Zhang L., Chu C., Lin X., Sun R., Li Z., Chen S., Liu Y., Wu J., Yu Z., Liu X. (2023). Tunable nanoparticles with aggregation-induced emission heater for precise synergistic photothermal and thermodynamic oral cancer therapy of patient-derived tumor xenograft. Adv. Sci..

[31] Zhuang Z., Bu F., Luo W., Peng H., Chen S., Hu R., Qin A., Zhao Z., Tang B.Z. (2017). Steric, conjugation and electronic impacts on the photoluminescence and electroluminescence properties of luminogens based on phosphindole oxide. J. Mater. Chem. C.

[32] Du X., Su H., Zhao L., Xing X., Wang B., Qiu D., Wang J., Yuan M.-S. (2021). AIE-based donor–acceptor–donor fluorenone compound as multi-functional luminescence materials. Mater. Chem. Front..

[33] Yang M.Q., Deng J.R., Su H.F., Gu S.X., Zhang J., Zhong A.G., Wu F.S. (2021). Small organic molecule-based nanoparticles with red/near-infrared aggregation-induced emission for bioimaging and PDT/PTT synergistic therapy. Mater. Chem. Front..

[34] Su H., Xie T., Liu Y.U., Cui Y., Wen W., Tang B.Z., Qin W. (2023). Facile synthesis of ultrabright luminogens with specific lipid droplets targeting feature for in vivo two-photon fluorescence retina imaging. Chin. Chem. Lett..

[35] Li J., Dai J., Zhuang Z., Meng Z., Hu J.-J., Lou X., Xia F., Zhao Z., Tang B.Z. (2022). Combining PD-L1 blockade with immunogenic cell death induced by AIE photosensitizer to improve antitumor immunity. Biomaterials.

[36] Kroemer G., Galassi C., Zitvogel L., Galluzzi L. (2022). Immunogenic cell stress and death. Nat. Immunol..

[37] Zhao Z., Su H.F., Zhang P.F., Cai Y.J., Kwok R.T.K., Chen Y.C., He Z.K., Gu X.G., He X.W., Sung H.H.Y., Willimas I.D., Lam J.W.Y., Zhang Z.F., Tang B.Z. (2017). Polyyne bridged AIE luminogens with red emission: design, synthesis, properties and applications. J. Mater. Chem. B.

[38] Li Y., Peng Q., Li S., Cai Y., Zhang B., Sun K., Ma J., Yang C., Hou G.F. (2019). A multifunctional quinoxalin-based AIEgen used for fluorescent thermo-sensing and image-guided photodynamic therapy. Sens. Actuators B Chem..

[39] Lyublinskaya O.G., Ivanova J.S., Pugovkina N.A., Kozhukharova I.V., Kovaleva Z.V., Shatrova A.N., Aksenov N.D., Zenin V.V., Kaulin Y.A., Gamaley I.A., Nikolsky N.N. (2017). Redox environment in stem and differentiated cells: a quantitative approach. Redox Biol..

[40] Chen D., Xu Q., Wang W., Shao J., Huang W., Dong X. (2021). Type I photosensitizers revitalizing photodynamic oncotherapy. Small.

[41] Zhuang Z., Dai J., Yu M., Li J., Shen P., Hu R., Lou X., Zhao Z., Tang B.Z. (2020). Type I photosensitizers based on phosphindole oxide for photodynamic therapy: apoptosis and autophagy induced by endoplasmic reticulum stress. Chem. Sci..

[42] Li J., Zhuang Z., Zhao Z., Tang B.Z. (2022). Type I AIE photosensitizers: mechanism and application. View.

[43] Lu Y., Li L., Lin Z., Li M., Hu X., Zhang Y., Peng M., Xia H., Han G. (2018). Enhancing osteosarcoma killing and CT imaging using ultrahigh drug loading and NIR-responsive bismuth Sulfide@Mesoporous silica nanoparticles. Adv. Healthcare Mater..

[44] Wojtoniszak M., Roginska D., Machalinski B., Drozdzik M., Mijowska E. (2013). Graphene oxide functionalized with methylene blue and its performance in singlet oxygen generation. Mater. Res. Bull..

[45] Zhao B., Yin J.-J., Bilski P.J., Chignell C.F., Roberts J.E., He Y.-Y. (2009). Enhanced photodynamic efficacy towards melanoma cells by encapsulation of Pc4 in silica nanoparticles. Toxicol. Appl. Pharmacol..

[46] Zhuang J., Ma Z., Li N., Chen H., Yang L., Lu Y., Guo K., Zhao N., Tang B.Z. (2024). Molecular engineering of plasma membrane and mitochondria dual-targeted NIR-II AIE photosensitizer evoking synergetic pyroptosis and apoptosis. Adv. Mater..

[47] Yang H., Li F., Chen S., Jin S., Sun W., Wei L., Chen W., Xu G., Song W., Zhong W. (2024). Endoplasmic reticulum and mitochondrial double-targeted NIR photosensitizer synergistically promote tumor cell death. Mater. Des..

[48] Wang S., Liao Y., Wu Z., Peng Y., Liu Y., Chen Y., Shao L., Zeng Z., Liu Y. (2023). A lysosomes and mitochondria dual-targeting AIE-active NIR photosensitizer: constructing amphiphilic structure for enhanced antitumor activity and two-photon imaging. Mater. Today Bio.

[49] Chen X., Li Y., Li S., Gao M., Ren L., Tang B.Z. (2018). Mitochondria- and lysosomes-targeted synergistic chemo-photodynamic therapy associated with self-monitoring by dual light-up fluorescence. Adv. Funct. Mater..

[50] Obeid M., Panaretakis T., Tesniere A., Joza N., Tufi R., Apetoh L., Ghiringhelli F.o., Zitvogel L., Kroemer G. (2007). Leveraging the immune system during chemotherapy: moving calreticulin to the cell surface converts apoptotic death from “silent” to immunogenic. Cancer Res..

[51] Garg A.D., Krysko D.V., Verfaillie T., Kaczmarek A., Ferreira G.B., Marysael T., Rubio N., Firczuk M., Mathieu C., Roebroek A.J., Annaert W., Golab J., de Witte P., Vandenabeele P., Agostinis P. (2012). A novel pathway combining calreticulin exposure and ATP secretion in immunogenic cancer cell death. EMBO J..

[52] Manganelli V., Capozzi A., Truglia S., Alessandri C., Lococo E., Garofalo T., De Carolis C., Conti F., Valesini G., Sorice M., Longo A., Misasi R. (2017). Elevated serum level of HMGB1 in patients with the antiphospholipid syndrome. J. Immunol. Res.

[53] Kepp O., Bezu L., Yamazaki T., Di Virgilio F., Smyth M.J., Kroemer G., Galluzzi L. (2021). ATP and cancer immunosurveillance. EMBO J..

[54] Pandolfi J.B., Ferraro A.A., Sananez I., Gancedo M.C., Baz P., Billordo L.A., Fainboim L., Arruvito L. (2016). ATP-induced inflammation drives tissue-resident Th17 cells in metabolically unhealthy obesity. J. Immunol..

[55] Medina C.B., Mehrotra P., Arandjelovic S., Perry J.S.A., Guo Y., Morioka S., Barron B., Walk S.F., Ghesquière B., Krupnick A.S., Lorenz U., Ravichandran K.S. (2020). Metabolites released from apoptotic cells act as tissue messengers. Nature.

