# Supplementary material for: Dual-organelle targeted photosensitizer with AIE characteristics for triple-negative breast cancer photodynamic therapy via apoptosis and immunogenic cell death
Source: Mater Today Bio. 2025 May 4;32:101828. doi: 10.1016/j.mtbio.2025.101828 (PMC12134542; doi:10.1016/j.mtbio.2025.101828)
Supplement: Multimedia component 1 [file mmc1.pdf]

Supporting Information

Dual-Organelle Targeted Photosensitizer with AIE Characteristics for Triple-negative Breast Cancer Photodynamic Therapy via Apoptosis and Immunogenic Cell Death

Wei Wen,[a,1] Jianqing Li,[b,1] Wenzhao Shang,[a,1] Zeyan Zhuang,[b] Xiepeng Deng,[a] Xueke Yan,[a] Dalu Xie,[a] Chen Cui,[a] Zujin Zhao,[b,]* Ben Zhong Tang, [c,]* and Huifang Su[a,]*

[a] Department of Orthopaedic Surgery, The First Affiliated Hospital of Zhengzhou University, Zhengzhou, Henan, 450052, PR China.

[b] State Key Laboratory of Luminescent Materials and Devices, Guangdong Provincial Key Laboratory of Luminescence from Molecular Aggregates, South China University of Technology, Guangzhou 510640, China.

[c] School of Science and Engineering, Shenzhen Institute of Aggregate Science and Technology, The Chinese University of Hong Kong, Shenzhen, Guangdong 518172, China.

* Corresponding author.

Huifang Su: suhuif@mail2.sysu.edu.cn

Zujin Zhao: mszjzhao@scut.edu.cn

Ben Zhong Tang: tangbenz@cuhk.edu.cn

[1] These authors contributed to the work equally.

1 Materials and instruments

1.1 Materials

Phosphate buffered saline (PBS), fetal bovine serum (FBS), penicillin, streptomycin, and Cell culture medium were acquired from Servicebio. 2’,7’-dichlorofluorescin diacetate (DCFH-DA) and Hydroxyphenyl Fluorescein (HPF) were acquired from Aladdin. 9,10-Anthracenediyl-bis (methylene) dimalonic acid (ABDA) was purchased from Sigma-Aldrich. 3-(4,5-Dimethyl-2-thiazolyl)-2,5-

diphenyl-2H-tetrazolium bromide (MTT) was acquired from J&K Scientific Ltd. Commercial dyes ER-Tracker Red, Mito-Tracker Red, Lyso-Tracker Red, and DAPI were acquired from Thermo Fisher Scientific. Calcein-Am and propidium iodide (PI) were acquired from Beyotime. Antibodies (anti-PARP, anti-Caspase-3, anti-Bcl-2) were purchased from Cell Signaling Technology. Antibodies (anti-CRT, anti-HMGB1, anti-β-Actin) were purchased from Proteintech. ECL chemiluminescence reagent kit was acquired from EpiZyme. MG-63, 4T1 cells were acquired from Procell Life Science & Technology Co, Ltd.

1.2 Instruments

^{1}H and ^{13}C NMR spectra were tested on a Bruker AV 400 or 500 spectrometers in deuterated methylene dichloride, chloroform, or dimethyl sulfoxide using tetramethylsilane (TMS; $\delta = 0$) as an internal reference at room temperature. High-resolution mass spectra (HRMS) were recorded on Agilent1290/ Bruker maxis impact mass spectrometer operating in positive mode. UV-vis absorption spectra were measured on a SHIMADZU UV-2600 spectrophotometer. Photoluminescence (PL) spectra were recorded on a Horiba Fluoromax-4 fluorescence spectrophotometer. Particle size analysis was performed on a Malvern Zetasizer Nano-S90. Confocal laser scanning microscopy (CLSM) images were obtained on a Zeiss LSM 980 Laser Scanning Confocal Microscope. The cell viability was analyzed by a microplate reader (Tecan Infinite M200PRO). The white light source was the CXE-350 Xenon Fiber Optic Lighting System manufactured by Beijing Normal University Optoelectronic Instrument Factory. The irradiated light energy density was measured with an FZ-A irradiance meter manufactured by Beijing Normal University Photoelectric Instrument Factory. Western blot was imaged using the Amersham Imager 680. The AniView100 Multimode Live Animal Imaging System measured the fluorescence intensity of mouse tumor tissues.

2 Experimental Methods

2.1 Syntheses and Characterization

Scheme S1 shows the synthetic route of 2TPA-PIMe. 2TPA-PIO was synthesized according to previous literature [1].

Synthesis of the intermediate compound (2TPA-PI):

A mixture of 2TPA-PIO (1.42 g, 2 mmol) and trichlorosilane (0.27 g, 4 mmol) in toluene (30 mL)

was stirred at room temperature for 2 h under nitrogen. The mixture was added to a saturated NaOH aqueous solution (5 mL) at 0 °C, and the resulting mixture was warmed to room temperature. After extraction, the solvent was evaporated under reduced pressure, and the residue was purified by column chromatography on silica gel by using petroleum ether/dichloromethane (5:1 v/v) as eluent to afford the product as a green solid (1.25 g, 90 %). ^{1}H NMR (400 MHz, DMSO), δ (TMS, ppm): 7.67 (dd, *J* = 7.5, 4.7 Hz, 1H), 7.47 – 7.15 (m, 18H), 7.13 – 6.98 (m, 12H), 6.94 (d, *J* = 8.1 Hz, 4H), 6.71 (d, *J* = 8.7 Hz, 2H). ^{13}C NMR (100 MHz, DMSO), δ (TMS, ppm): 147.46, 147.35 (d, *J* = 5.0 Hz), 147.11, 146.49, 144.67 (d, *J* = 5.0 Hz), 144.14 (d, *J* = 9.0 Hz), 142.50, 133.86, 133.69, 133.28 (d, *J* = 19.0 Hz), 131.11, 130.87, 130.77, 130.55, 130.19, 130.02, 130.00, 129.59, 129.38 (d, *J* = 8.0 Hz), 128.96, 126.58, 124.97, 124.53, 123.99, 123.87, 123.68, 121.81.

Synthesis of 2TPA-PIMe:

A mixture of 2TPA-PI (1.25 g, 2 mmol) and methyl trifluoromethanesulfonate (1.31 g, 8 mmol) in 20 mL dichloromethane and then stirred at room temperature for 12 h under nitrogen. After solvent evaporation, the residue was purified via silica gel column chromatography using dichloromethane/ethyl acetate as eluent. The red solid of 2TPA-PIMe in a 75% yield (1.07 g) was obtained. ^{1}H NMR (500 MHz, CD2Cl2), δ (TMS, ppm): 8.05 (dd, J = 10.9, 6.8 Hz, 1H), 7.89 – 7.77 (m, 4H), 7.71 – 7.60 (m, 4H), 7.36 – 7.24 (m, 10H), 7.18 – 7.06 (m, 13H), 6.80 (s, 4H), 2.65 (d, J = 14.0 Hz, 3H). ^{13}C NMR (125 MHz, CD2Cl2), δ (TMS, ppm): ^{13}C NMR (126 MHz, Methylene Chloride-d2) δ 156.82 (d, J = 20.0 Hz), 150.90, 150.17, 147.89 (d, J = 35.0 Hz), 137.01, 136.98, 136.95, 133.43 (d, J = 11.25 Hz), 132.93 (d, J = 10.0 Hz), 132.08 (d, J = 13.75 Hz), 132.06, 131.97, 131.56, 131.11 (d, J = 6.25 Hz), 130.88 (d, J = 2.50 Hz), 127.80 (d, J = 11.25 Hz), 127.02, 126.90, 125.78, 125.63, 124.28, 124.17, 123.15 (d, J = 11.25 Hz), 122.56, 122.03, 120.94, 118.67, 118.01, 6.82 (d, J = 26.25 Hz). HRMS: m/z calcd for C51H40N2P+: 711.2924 [M+]; found: 711.2948.

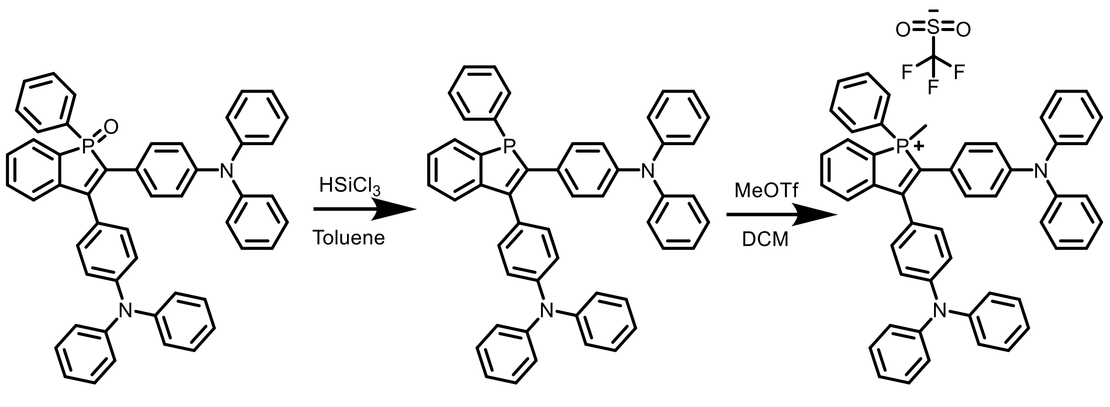

Scheme S1. The synthetic route of 2TPA-PIMe.

2.2 ROS generation detection in aqueous solution

Detection of general ROS in aqueous solution: Using 2',7'-Dichlorodihydrofluorescein (DCFH) as an indicator. Using standard procedures, 2',7'-Dichlorodihydrofluorescein diacetate (DCFH-DA) was initially converted to DCFH. The indicator in PBS was subsequently diluted to 10 μM in a solution of 2TPA-PIMe (1 μM). The solution was subsequently exposed to white light (20 mW/cm^2) to assess its fluorescence intensity. The fluorescence signal reception range is 510 to 560 nm, utilizing 504 nm as the excitation wavelength. The production of active oxygen was monitored using the variation in fluorescence intensity at 525 nm.

Detection of $^1O_2$ in aqueous solution: The reduction in the ABDA absorbance spectrum at 380 nm was quantified to signify the rate of $^1O_2$ production. The production of $^1O_2$ in 2TPA-PIMe aqueous solution utilizing 9,10-dimethyl-bis(methylene)bismalonic acid (ABDA) as an indicator when exposed to white light (20 mW/cm^2). And 1 μM 2TPA-PIMe solution was used to dilute the indicator to 10 μM. The indicator's absorption spectrum spans from 330 to 450 nm, while its excitation wavelength is 480 nm. The drop in the ABDA absorbance spectra at 380 nm calculates the production rate of $^1O_2$.

Detection of hydroxyl radicals (·OH) in aqueous solution: The production of ·OH in 2TPA-PIMe aqueous solution under white light irradiation was measured using hydroxyphenyl fluorescein (HPF) as an indicator. After diluting the indicator with PBS to 5 μM, 1 μM of 2TPA-PIMe solution was added. The excitation wavelength was 480 nm, while the fluorescence signal's receiving range was 500–550 nm. The rate at which hydroxyl radicals (·OH) are generated was determined by measuring the change in fluorescence intensity at 514 nm.

2.3 Cell culture

MG-63 cells (human osteosarcoma) were cultivated in DMEM media; 4T1 cells (mouse mammary carcinoma) were cultivated in RPMI 1640 media. All aforementioned culture media were augmented with FBS (10%) and penicillin-streptomycin (1%). The cell culture conditions were 37°C and 5% CO^2.

.

2.4 Cellular uptake of 2TPA-PIMe

MG-63 cells were inoculated in confocal culture dishes, with a density of 1×10^5 cells per dish. They were then incubated for 24 h. Subsequently, they were exposed to a media containing 1μM 2TPA-PIMe for 0.5, 1, 2, 4, 10, and 24 hours. The cells were subsequently analyzed with CLSM.

2.5 Organelles co-localization imaging

MG-63 and 4T1 cells were cultured in a confocal dish for 24 hours at 37°C. Initially, cells were cultured in media supplemented with 1 μM 2TPA-PIMe for 4 hours, followed by 30-minute staining with commercially available dyes: Mito-Tracker Red (working concentration: 100 nM), ER-Tracker Red (working concentration: 1 μM), and Lyso-Tracker Green (working concentration: 100 nM). Following three rinses with PBS, the cells were photographed using CLSM. The following are the spectrum conditions of CLSM: for 2TPA-PIMe, λex=488 nm, and λem=590-720 nm; for Mito-Tracker Red and ER-Tracker Red, λex=543 nm and λem=590-720 nm; for Lyso-Tracker Green, λ_{ex}=488 nm and λ_{em}=490-530 nm.

2.6 Cytotoxicity evaluation of cancer cells induced by PDT

The MTT test was employed to assess the cytotoxicity of 2TPA-PIMe in both dark and light conditions. MG-63 and 4T1 cells were initially plated in 96-well plates with a density of 1×10^4 cells per well and incubated for 24 hours. The cells were treated with varying doses of 2TPA-PIMe in a fresh medium for 4 hours. Afterward, the cells were irradiated with white light at 40 mW/cm^2 for 10 minutes. The cells were subsequently cultured at 37 °C for 24 hours. Simultaneously, a dark cytotoxicity investigation was performed on the 2TPA-PIMe incubated cells without light irradiation. Following a 24-hour incubation, the cells were exposed to fresh serum-free media enriched with 10% MTT for 4 hours without light. Subsequently, all media were eliminated, and 100 μl of DMSO was added. The absorbance at 570 nm was quantified using a microplate reader to represent cell viability.

2.7 Intracellular ROS generation test

The cells were incubated in confocal culture dishes for 24 hours. The cells underwent further treatment with 2TPA-PIMe (1 μM) in fresh media for 4 hours. The cells underwent treatment with 10 μM DCFH-DA for 30 minutes. The cells were washed three times with PBS and subjected to white light (40 mW/cm^2) for 5 minutes, after which CLSM imaging was conducted immediately. The cells subjected to 2TPA-PIMe without white light exposure and PBS with or without white light exposure functioned as the controls. Utilize CLSM to capture images of cells. λ_{ex}=488 nm and λ_{em}=493-550 nm.

2.8 Live/dead cell co-staining assays

The cells were incubated in confocal culture dishes for 24 hours. After that, the cells underwent further treatment with 2TPA-PIMe (1 μM) in fresh media for 4 hours, irradiating with 40 mW/cm^2 white light for 10 minutes. The cells were then grown at 37 °C for 24 hours. The cells exposed to 2TPA-PIMe without white light and those treated with PBS with or without white light served as the controls. Then, CLSM imaging was performed using the Calcein/PI Cell Viability and Cytotoxicity Assay Kit (Purchased from Beyotime).

2.9 Western blot analysis

The soluble protein fraction was acquired using centrifugation and subsequently isolated polyacrylamide gel electrophoresis. After that, The proteins were then transferred to a PVDF membrane and blocked with skim milk powder. The subsequent primary antibodies (dilutions) were incubated overnight at 4°C. Following a washing step and contingent upon the primary antibody employed, the blot was incubated with a secondary antibody for one hour at ambient temperature. The blot was subsequently examined with an ECL chemiluminescence kit and quantified with ImageJ software.

2.10 Immunofluorescence images

Cells were cultured in confocal dishes until confluence reached 80%. Cells were exposed to 1μM AIE in fresh media for 4 hours and then irradiated with 40mW/cm^2 white light for 10 minutes. The cells were subsequently incubated at 37°C for 4 hours. Cells subjected to 2TPA-PIMe without white light irradiation and cells treated with PBS, with or without white light irradiation, functioned as controls. The cells were initially subjected to a 15-minute treatment with 4% (v/v) paraformaldehyde, followed by a 10-minute exposure to 0.2% Triton X-100, and concluded with a 30-minute incubation

in 5% bovine serum albumin (BSA). All aforementioned activities were conducted at ambient temperature. Cells were subsequently incubated with anti-CRT and anti-HMGB1 antibodies overnight at 4°C. The following three washes were done with phosphate-buffered saline containing Tween-20 (PBS-T), and the cells were incubated with the secondary antibody for one hour. Subsequently, the cells were washed three times. Subsequently, cell images were observed using CLSM. λ_{ex}=488 nm and λ_{em}=490-530nm.

2.11 Detection of extracellular ATP

The cells were seeded into cell culture dishes until the confluence reached 80%. Cells were exposed to 1μM 2TPA-PIMe in fresh media for 4 hours and then irradiated with 40mW/cm^2 white light for 10 minutes. The cells were subsequently incubated at 37°C for 4 hours. Cells subjected to 2TPA-PIMe without exposure to white light and cells treated with PBS, with or without white light irradiation, functioned as controls. Supernatants were obtained, and the concentration of released ATP was quantitatively measured using an ATP bioluminescence assay kit.

2.12 Animals and Subcutaneous Tumor Model

All animals were maintained at 25°C and 55% humidity, with unlimited access to standard laboratory water and food. Subcutaneous tumor models were created by injecting 5 × 10^6 4T1 cells suspended in 100 μL of PBS into the right flank of each 5-week-old male Balb/C mouse. Upon the tumor volume attaining 100–150 mm^3, animals underwent assessment for in vivo imaging and photodynamic therapy. Tumors were assessed utilizing the formula: tumor volume (mm^3) = (width^2 × length) × 0.5.

2.13 In vivo imaging

Upon the tumor volume reaching approximately 150 mm^3, 2TPA-PIMe (5 mg/kg, 50 μL) was administered via intratumoral injection. Following injection, the fluorescence intensity in the tumor tissue was measured at various time intervals (λ_{ex}=465 nm, λ_{em}=640 nm) utilizing the AniView100 multi-mode live animal imaging system, and the resultant data were analyzed with AniView software.

2.14 In vivo PDT evaluation

The mice were randomly allocated into four groups (n = 5): PBS, PBS + Light (PBS + L), 2TPA-

PIMe, and 2TPA-PIMe + Light (2TPA-PIMe + L). Upon the tumors attaining a volume of approximately 150 mm^3, 2TPA-PIMe (5 mg/kg) and PBS were administered intratumorally. Following 24 hours post-injection, the tumor regions of the "+ Light" groups are subjected to white light (200 mW/cm^2) for 30 minutes. The treatment is administered every three days for a total of three cycles. The body weight and tumor volume of the mice were assessed bi-daily.

2.15 Histological analysis

Upon the tumor volume reaching roughly 1000 mm^3, all mice were euthanized, and the tumors were resected and weighed. The tumors were subsequently submerged in 4% v/v formalin overnight, paraffin slices were produced, and hematoxylin and eosin staining were conducted.

2.16 In vivo biosafety evaluation

Before euthanasia, the ocular globes of the mice were excised, and blood specimens were obtained in collecting tubes. The primary hematological indices and serum biochemical markers were subsequently examined. Concurrently, the principal organs (heart, liver, spleen, lung, kidney, and intestine) were excised from the mice and subsequently analyzed using H&E staining.

2.17 Statistical analysis

Data ($n \geqslant 3$) are presented as mean ± standard deviation (SD). One-way analysis of variance (ANOVA) was used to assess significance, utilizing GraphPad Prism 7. Statistical significance was established as *P<0.05; **P<0.01; ***P<0.001; ****P<0.0001; not significant, *ns*.

3 Supplementary figures and tables

A

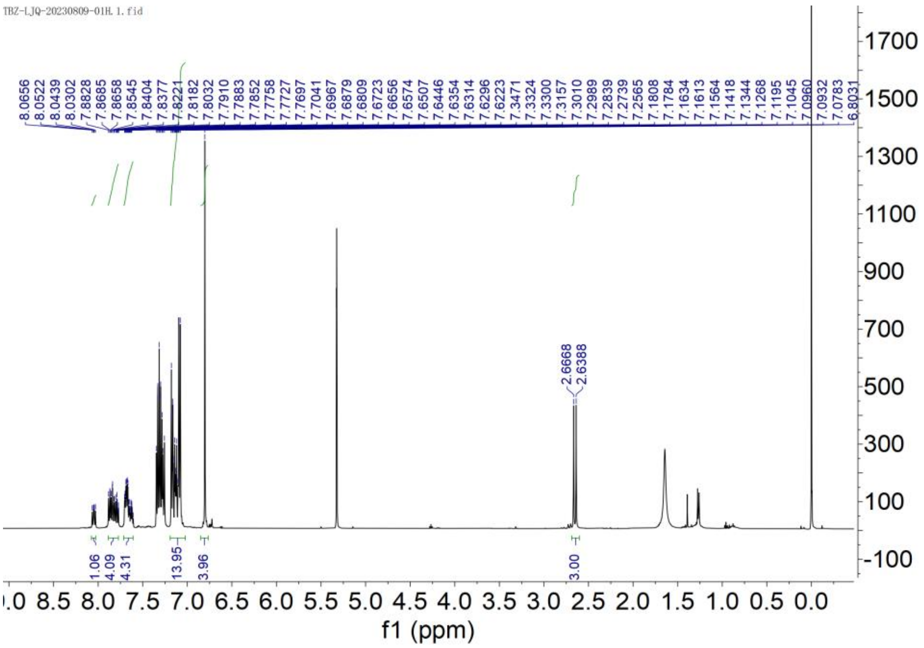
TBZ-LJQ-20230809-01H.1.fid
8.0656 8.0522 8.0439 8.0302 7.8828 7.8685 7.8658 7.8545 7.8404 7.8377 7.8221 7.8182 7.8032 7.7910 7.7883 7.7852 7.7758 7.7727 7.7697 7.7041 7.6967 7.6879 7.6809 7.6723 7.6656 7.6574 7.6507 7.6446 7.6354 7.6314 7.6296 7.6223 7.3471 7.3324 7.3300 7.3157 7.3010 7.2989 7.2839 7.2739 7.2565 7.1808 7.1784 7.1634 7.1613 7.1564 7.1418 7.1344 7.1268 7.1195 7.1045 7.0960 7.0932 7.0783 6.8031
2.6668 2.6388
1.06 4.09 4.31 13.95 3.96 3.00
f1 (ppm)

B

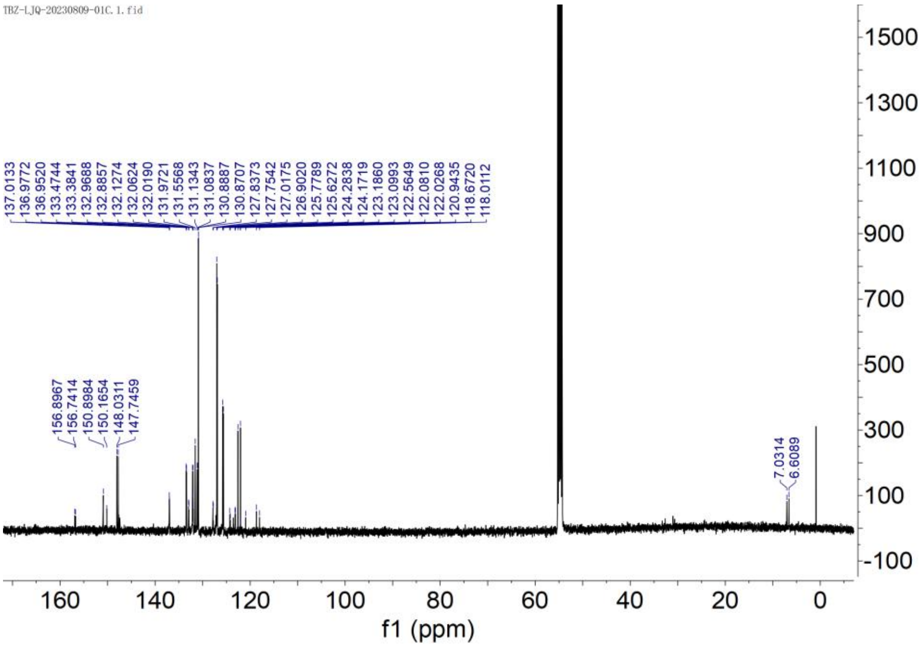
TBZ-LJQ-20230809-01C.1.fid
137.0133 136.9772 136.9520 133.4744 133.3841 132.9688 132.8857 132.1274 132.0624 132.0190 131.9721 131.5568 131.1343 131.0837 130.8887 130.8707 127.8373 127.7542 127.0175 126.9020 125.7769 125.6272 124.2838 124.1719 123.1860 123.0993 122.5649 122.0810 122.0268 120.9435 118.6720 118.0112
156.8967 156.7414 150.8984 150.1654 148.0311 147.7459
7.0314 6.6089
f1 (ppm)

C

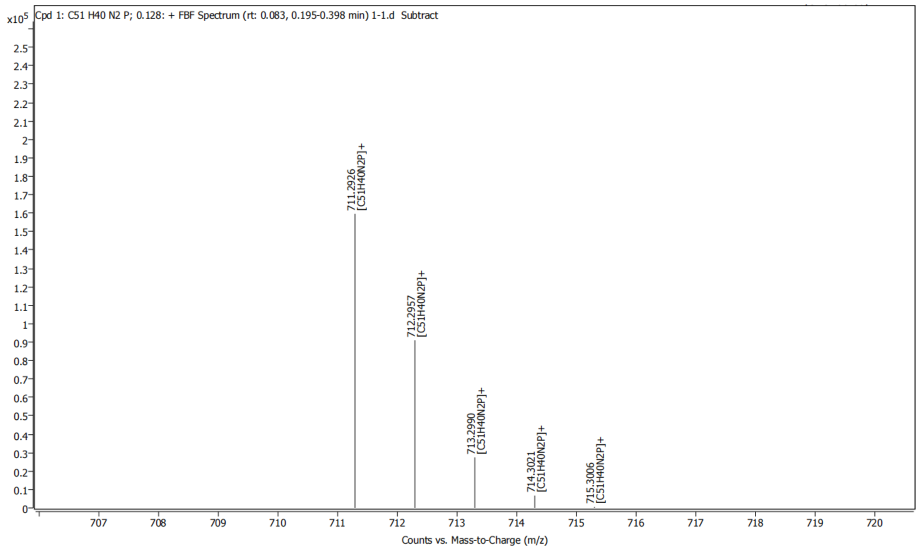
Cpd 1: C51 H40 N2 P; 0.128: + FBF Spectrum (rt: 0.083, 0.195-0.398 min) 1-1.d Subtract
x10^5
711.2926 [C51H40N2P]+
712.2957 [C51H40N2P]+
713.2990 [C51H40N2P]+
714.3021 [C51H40N2P]+
715.3006 [C51H40N2P]+
Counts vs. Mass-to-Charge (m/z)

Figure S1. (A) ^{1}H NMR, (B) ^{13}C NMR and (C)mass spectrum of 2TPA-PIMe.

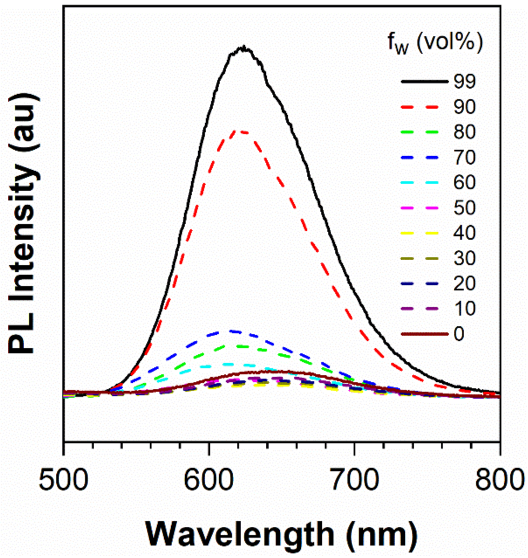

Figure S2. PL spectra of 2TPA-PIMe in DMSO/water mixtures with different f_ws (10 µM).

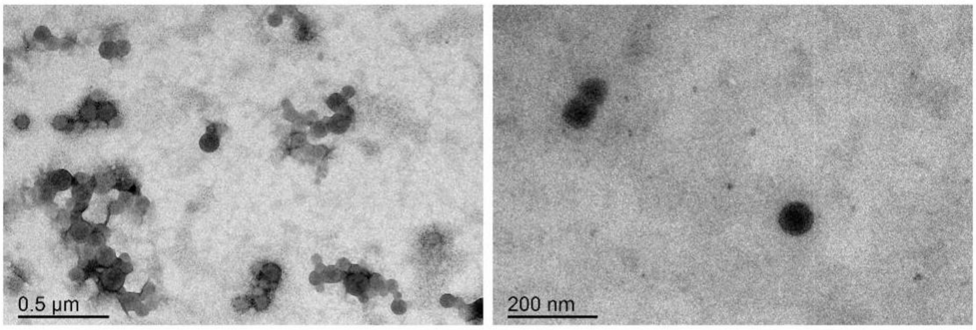

Figure S3. Transmission electron microscopy images for the kiln-dried films from the water with 1 vol% DMSO of 2TPA-PIMe.

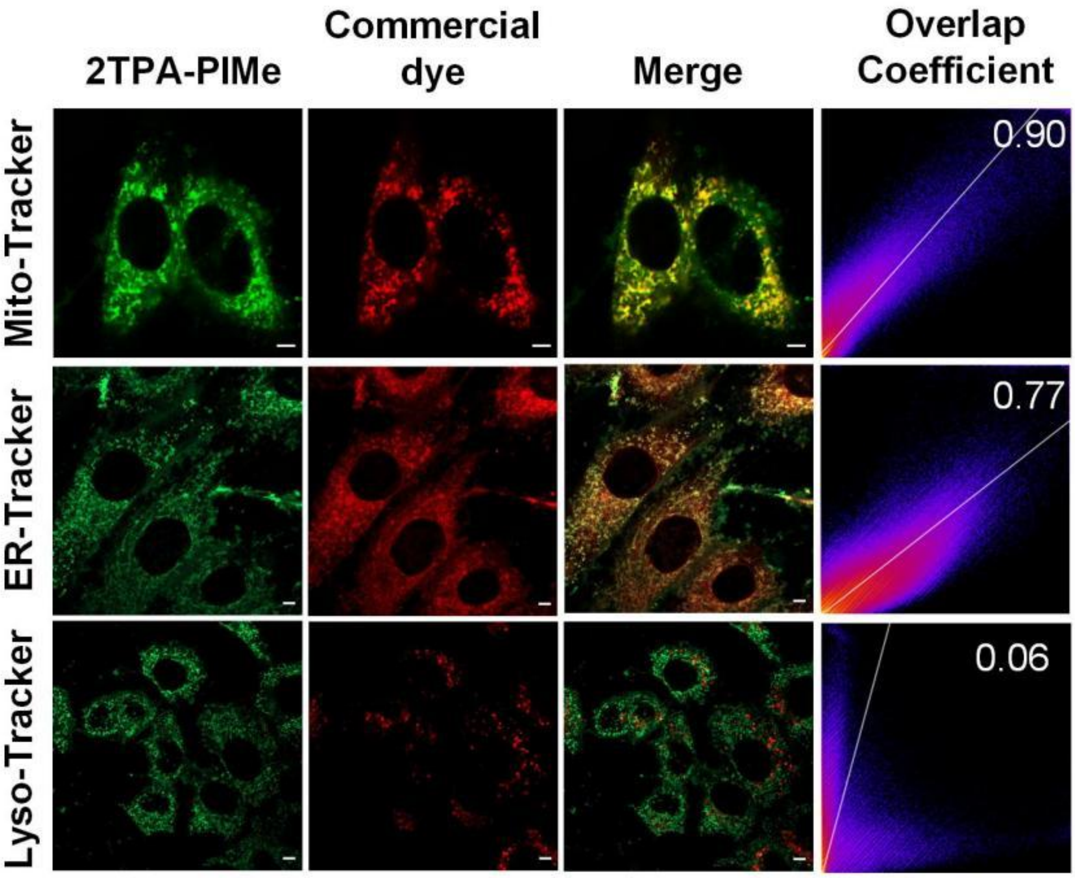

Figure S4. Colocalization images of 4T1 cells co-stained with 2TPA-PIMe (1 μM) and Mito-Tracker Red, ER-Tracker Red, and Lyso-Tracker Green. Scale bar: 5 μm.

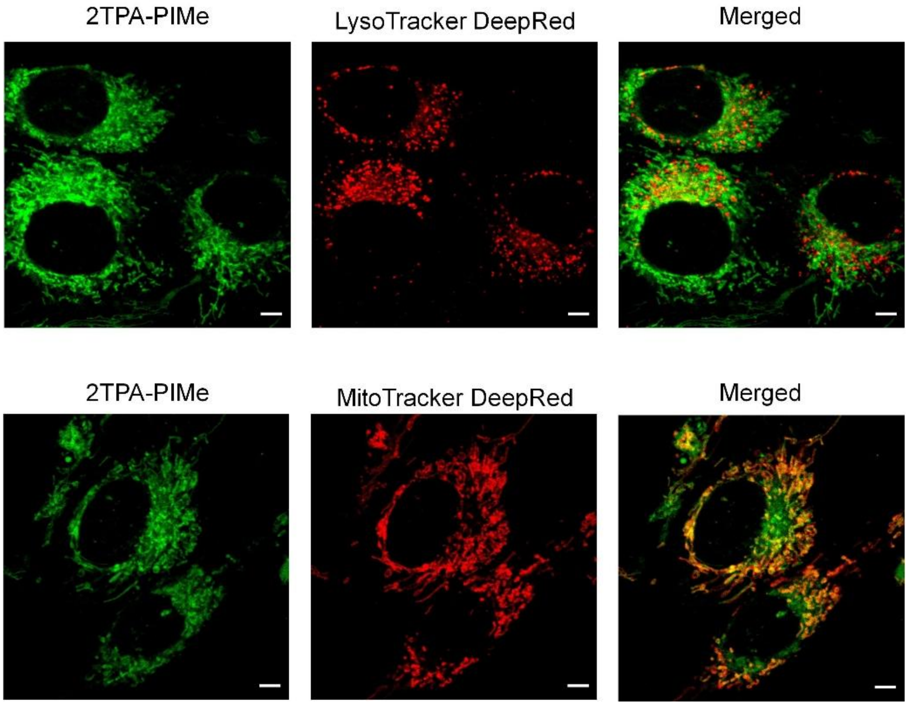

Figure S5. Colocalization images of MG63 cells co-stained with 2TPA-PIMe (1 μM) and Mito-Tracker DeepRed and Lyso-Tracker DeepRed. Scale bar: 5 μm.

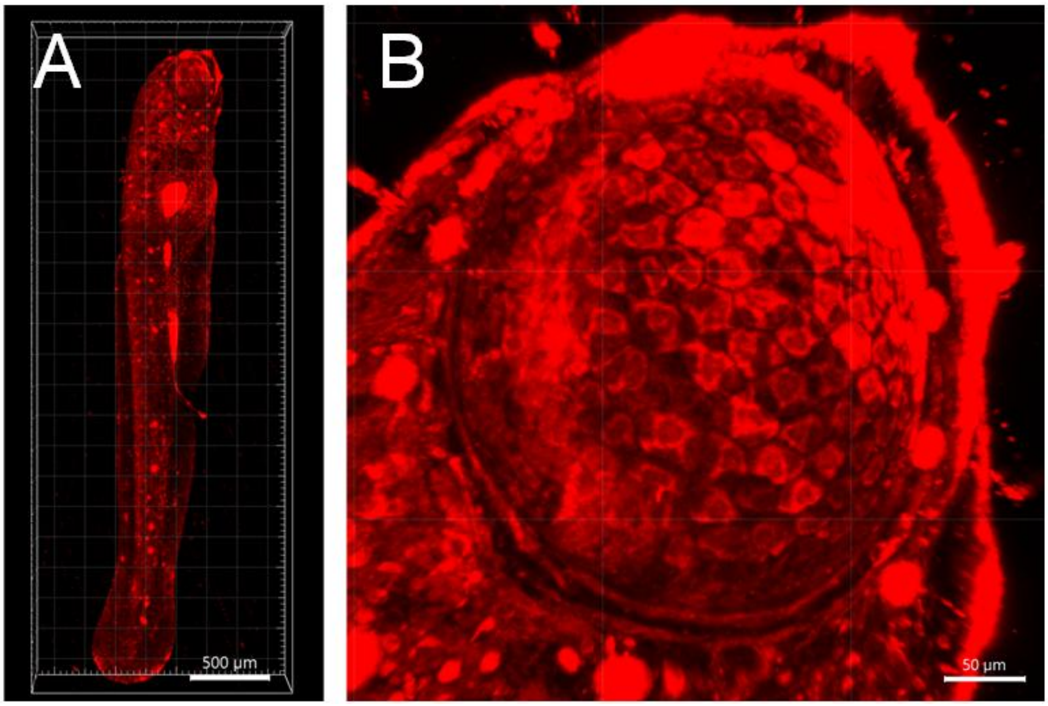

Figure S6. 3D reconstruction of one-photon CLSM images of Zebrafish embryo (A) and local magnified image of the eyeball (B) after staining with 2TPA-PIMe for 1 h (10 μM). The excitation wavelength laser is 488 nm. Emission wavelengths are 506–734 nm

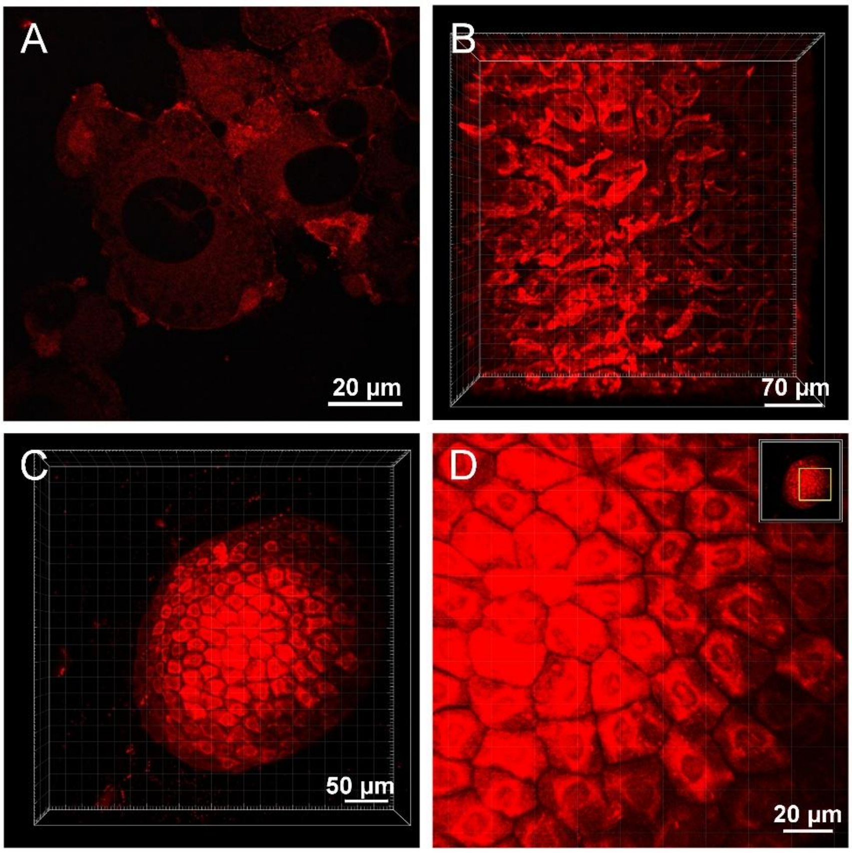

Figure S7. (A) Two-photon CLSM image of 4T1 cells stained with 2TPA-PIMe for 30 min (1 μM). (B) Two-photon CLSM image of mouse kidney frozen section treated with 2TPA-PIMe for 1 h (5 μM). (C) Two-photon CLSM image of zebrafish embryo eyeball and local magnified image (D) after staining with 2TPA-PIMe for 1 h (10 μM). The excitation wavelength for the two-photon laser excitation is 850 nm.

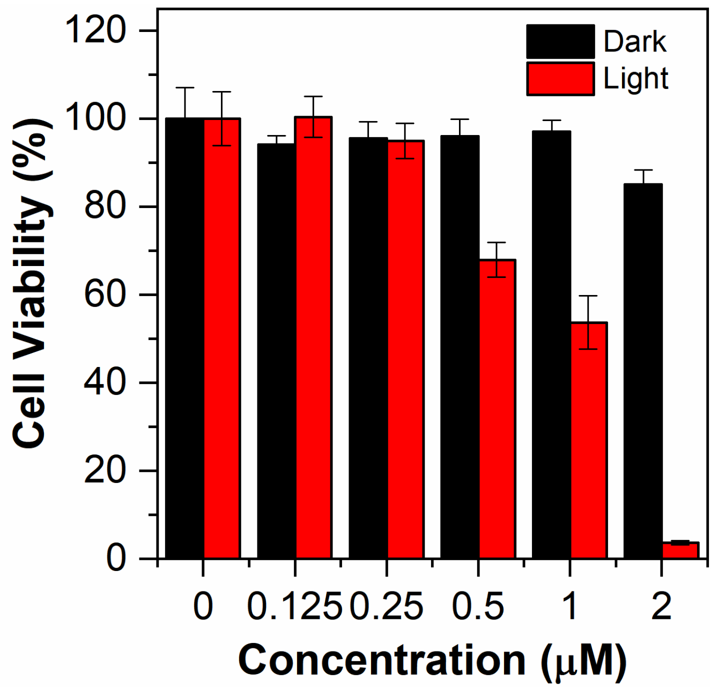

Figure S8. Cell viability of 4T1 cells after treatment with different concentrations of 2TPA-PIMe in the absence or presence of white light irradiation.

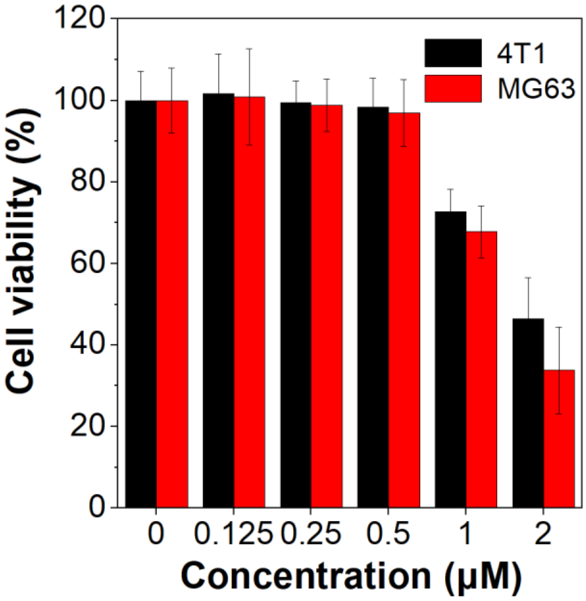

Figure S9. Cell viability of 4T1 and MG63 cells after treatment with different concentrations of 2TPA-PIMe and vitamin C (0.5mM)

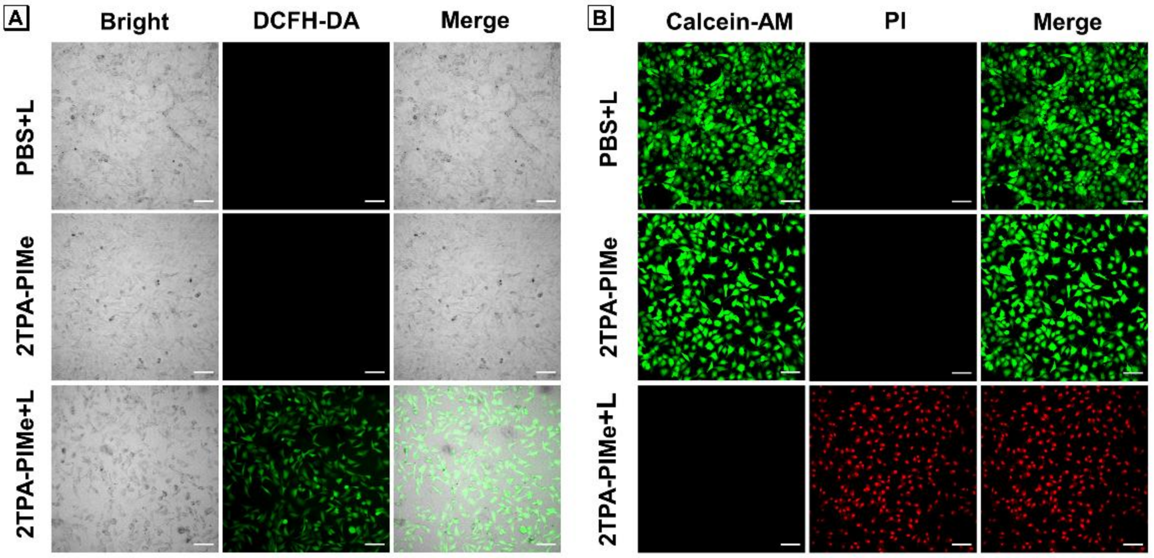

Figure S10. (A) General ROS generation of 2TPA-PIMe in 4T1 cells and (B) live/dead staining assay of 4T1 cells after treatment with PBS + Light (PBS + L), 2TPA-PIMe, 2TPA-PIMe + Light (2TPA-PIMe + L). (White light: 40 mW/cm^2 for 10 min). Scale bar: 100 μm.

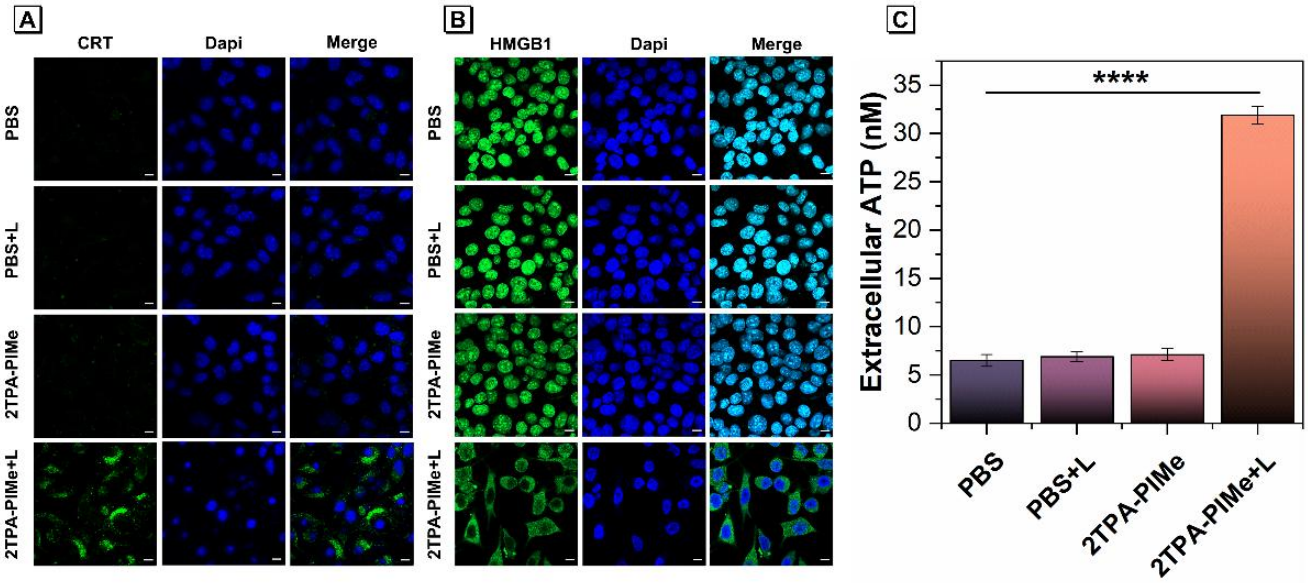

Figure S11. The immunofluorescence staining images of (A) CRT and (B) HMGB1 on 4T1 cells after treatment with PBS and 2TPA-PIMe (1 μM) with or without white light (40 mW cm^{-2}) for 10 min and then following incubation for 4 h. Scale bar: 10 μm. (C) Quantification of extracellular adenosine triphosphate (ATP) in 4T1 cells upon treatment with PBS or 2TPA-PIMe (1 μM) in the dark or upon irradiation (40 mW cm^{-2}). Data were shown as mean ± SD, n≥3. ($p < 0.05$, *; $p < 0.01$, **; $p < 0.001$, ***; $p < 0.0001$, ****; not significant, *ns*).

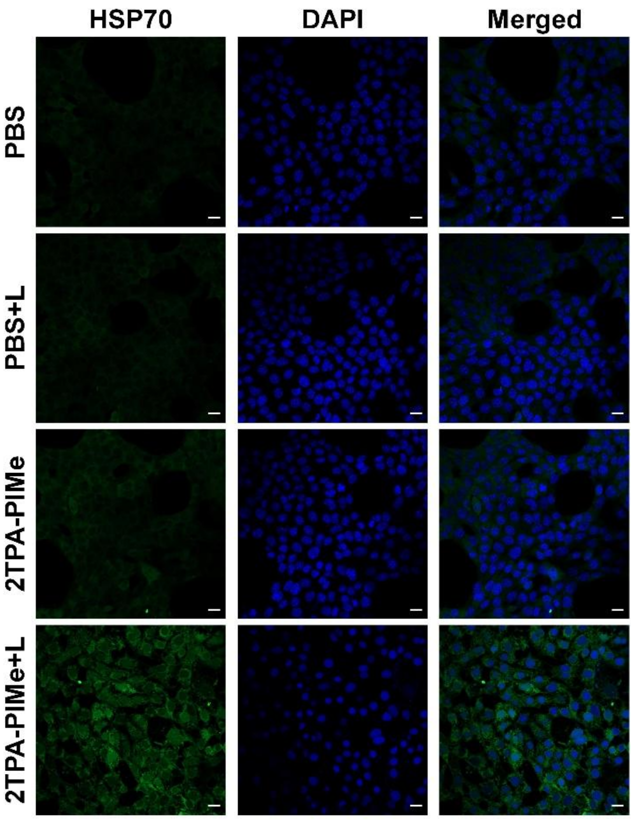

Figure S12. The immunofluorescence staining images of HSP70 on 4T1 cells after treatment with PBS and 2TPA-PIMe (1 μM) with or without white light (40 mW cm^{-2}) for 10 min and then following incubation for 4 h. Scale bar: 10 μm.

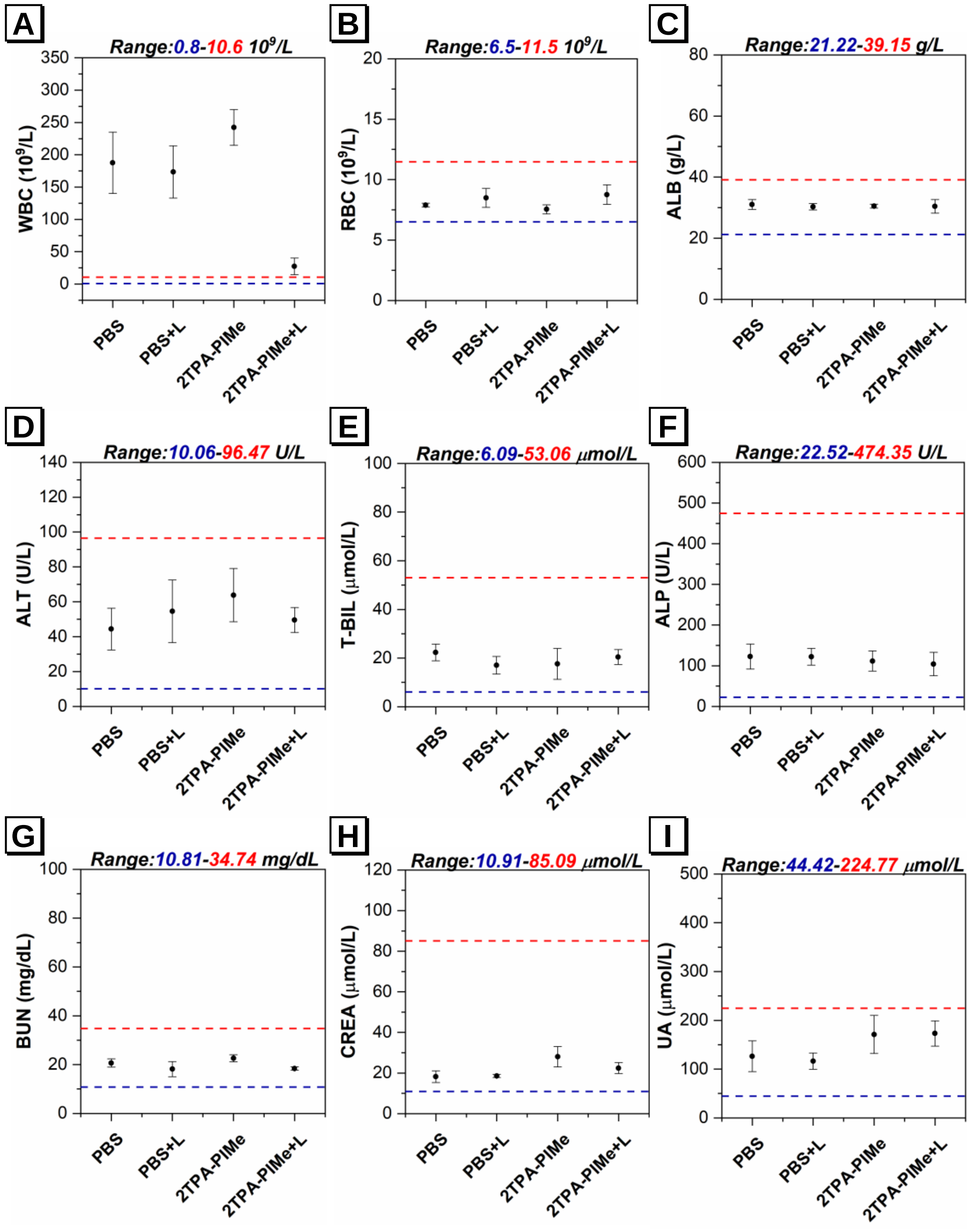

Figure S13. Blood routine and biochemical analysis results of (A) white blood cell (WBC), (B) red blood cell (RBC), (C) alanine albumin (ALB), (D) alanine aminotransferase (ALT), (E) total bilirubin (TBIL), (F) alkaline phosphatase (ALP), (G) blood urea nitrogen (BUN), (H) creatinine (CREA) and (I) uric acid (UA) in blood samples from mice after different treatments.

Table S1. Routine blood indexes of mice after different treatments.

		PBS	PBS+L	2TPA-PIMe	2TPA-PIMe+L	Reference Range
WBC	(10^9/L)	187.6±47.4	173.4±40.4	242.4±27.7	27.3±12.8	0.8-10.6
Lymph	(10^9/L)	77.5±30.7	51.7±43.5	63.1±67.7	17.9±6.69	0.6-8.9
Mon	(%)	11.9±5.32	8.30±2.68	11.5±2.70	1.07±0.69	0.04-1.4
Gran	(10^9/L)	92.9±56.2	113.3±78.1	167.7±50.6	8.32±11.8	0.23-3.6
RBC	(10^{12}/L)	7.89±0.15	8.48±0.78	7.54±0.37	8.76±0.81	6.5-11.5
HGB	(g/L)	131.2±5.85	137.3±12.5	129.0±2.64	131.7±10.9	110-165
HCT	(%)	39.0±1.00	42.1±4.42	37.1±1.43	43.4±3.06	35-55
MCV	(fL)	49.5±1.11	49.7±0.69	49.3±0.60	49.7±1.49	41-55
MCH	(pg)	16.5±0.72	16.1±0.20	17.1±0.75	15.0±0.21	13-18
MCHC	(g/L)	335.5±11.5	325.6±7.50	347.3±11.2	302.5±5.80	300-360
RDW	(%)	16.1±0.75	17.4±0.17	17.7±0.62	17.3±1.52	12-19
MPV	(fL)	5.87±0.27	5.83±0.40	5.96±0.05	5.75±0.45	4.0-6.2

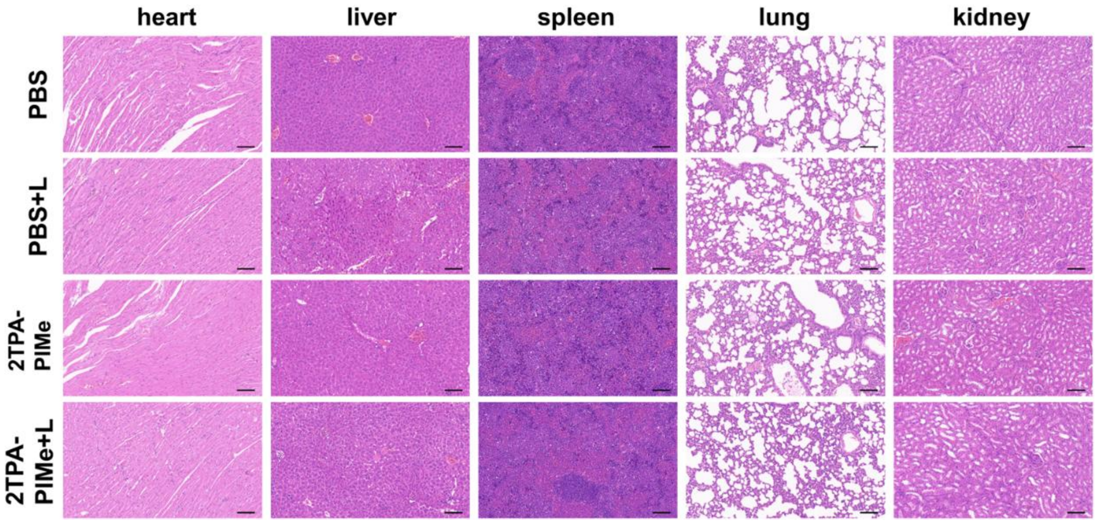

Figure S14. H&E-stained slice images of major organs after various treatments for 10 days. Scale bar: 100 μm.

References

[1] Z. Zhuang, F. Bu, W. Luo, H. Peng, S. Chen, R. Hu, A. Qin, Z. Zhao, B.Z. Tang, Steric, conjugation and electronic impacts on the photoluminescence and electroluminescence properties of luminogens based on phosphindole oxide, Journal of Materials Chemistry C 5(7) (2017) 1836-1842. https://doi.org/10.1039/C6TC05591E.
